# The Infantile Leukoencephalopathy-Associated Mutation of C11ORF73/HIKESHI Proteins Generates De Novo Interactive Activity with Filamin A, Inhibiting Oligodendroglial Cell Morphological Differentiation

**DOI:** 10.3390/medicines8020009

**Published:** 2021-02-01

**Authors:** Kohei Hattori, Kenji Tago, Shiori Memezawa, Arisa Ochiai, Sui Sawaguchi, Yukino Kato, Takanari Sato, Kazuma Tomizuka, Hiroaki Ooizumi, Katsuya Ohbuchi, Kazushige Mizoguchi, Yuki Miyamoto, Junji Yamauchi

**Affiliations:** 1 Laboratory of Molecular Neurology, Tokyo University of Pharmacy and Life Sciences, Hachioji, Tokyo 192-0392, Japan; ucqovp8er@gmail.com (K.H.); s167057@toyaku.ac.jp (S.M.); s169029@toyaku.ac.jp (A.O.); s167023@toyaku.ac.jp (S.S.); s179026@toyaku.ac.jp (Y.K.); s177030@toyaku.ac.jp (T.S.); 2 Department of Biochemistry, Jichi Medical University, Shimotsuke, Tochigi 321-0498, Japan; ktago@jichi.ac.jp; 3 Laboratory of Bioengineering, Tokyo University of Pharmacy and Life Sciences, Hachioji, Tokyo 192-0392, Japan; tomizuka@toyaku.ac.jp; 4 Tsumura Research Laboratories, Tsumura & Co., Inashiki, Ibaraki 200-1192, Japan; ooizumi_hiroaki@mail.tsumura.co.jp (H.O.); oka280928@gmail.com (K.O.); mizoguchi_kazushige@mail.tsumura.co.jp (K.M.); 5 Laboratory of Molecular Pharmacology, National Research Institute for Child Health and Development, Setagaya, Tokyo 157-8535, Japan; miyamoto-y@ncchd.go.jp

**Keywords:** leukoencephalopathy, HLD13, C11orf73, Filamin A, oligodendrocyte differentiation

## Abstract

**Background:** Genetic hypomyelinating diseases are a heterogeneous group of disorders involving the white matter. One infantile hypomyelinating leukoencephalopathy is associated with the homozygous variant (Cys4-to-Ser (C4S)) of the *c11orf7*3 gene. **Methods:** We observed that in mouse oligodendroglial FBD-102b cells, the C4S mutant proteins but not the wild type ones of C11orf73 are microscopically localized in the lysosome. And, they downregulate lysosome-related signaling in an immunoblotting technique. **Results:** The C4S mutant proteins specifically interact with Filamin A, which is known to anchor transmembrane proteins to the actin cytoskeleton; the C4S mutant proteins and Filamin A are also observed in the lysosome fraction. While parental FBD-102b cells and cells harboring the wild type constructs exhibit morphological differentiation, cells harboring C4S mutant constructs do not. It may be that morphological differentiation is inhibited because expression of these C4S mutant proteins leads to defects in the actin cytoskeletal network involving Filamin A. **Conclusions:** The findings that leukoencephalopathy-associated C11ORF73 mutant proteins specifically interact with Filamin A, are localized in the lysosome, and inhibit morphological differentiation shed light on the molecular and cellular pathological mechanisms that underlie infantile hypomyelinating leukoencephalopathy.

## 1. Introduction

Myelin sheaths are derived from the differentiated plasma membranes of oligodendroglial cells (oligodendrocytes) in the central nervous system (CNS) or Schwann cells in the peripheral nervous system (PNS). These cells wrap multiple layers of myelin, which then become the myelin sheaths, around the neuronal axons. These myelin sheaths grow dynamically to be more than 100 times larger than the collective surface area of the premyelinated plasma membranes [1,2,3,4]. Myelin sheaths play an indispensable role in the propagation of saltatory conduction. They also protect axons from physical and physiological stresses [1,2,3,4].

It is thought that defective myelin formation causes serious diseases. Hypomyelinating leukodystrophies (HLDs) are a recently classified group of hereditary neuropathies, primarily linked to oligodendrocytes, that affects 1 out of every 200,000 to 500,000 people. Pelizaeus-Merzbacher disease (PMD), now renamed HLD1, is a prototypic HLD [5,6,7,8]. Recent advances in powerful nucleotide sequencing technologies have enabled us to identify several unexpected genes that are responsible for other HLDs as well as other HLD-related neuropathies [5,6,7,8,9,10]. For example, HLD13 is caused by the Val54-to-Leu (V54L) mutation of cytoplasmic- and nuclear-localized C11ORF73 (also called HIKESHI) [10]. It is thought that C11ORF73 normally supports the heat-shock-induced nuclear transport of 70 kDa heat-shock proteins [11].

Furthermore, the infantile leukoencephalopathy-associated Cys4-to-Ser (C4S) mutation of C11ORF73 is known to decrease the protein expression levels, although C11ORF73 mutant proteins are still expressed at low levels in the nervous tissues [12]. To date, it has remained unclear whether or how these residual mutant proteins cause their pathological effects at the molecular and cellular levels.

Here, we report that the Cys4-to-Ser (C4S) mutant proteins of C11ORF73 are localized and aggregated in the lysosome in the mouse oligodendroglial FBD-102b cells that we have used as model oligodendrocytes. For comparison purposes, it is noteworthy that C11ORF73 Val54-to-Leu (V54L) mutant proteins are also associated with aggregation. Cells expressing the C4S mutant proteins exhibit decreased lysosomal mammalian target of Rapamycin (mTOR) signaling, including S6 and 4E-BP1 phosphorylation, which is essential for oligodendrocyte differentiation and myelination [1,2,3,4]. Additionally, the C4S mutant proteins, but not the wild type ones, interact with Filamin A, which anchors transmembrane proteins to the actin cytoskeleton and connects the actin filaments [13]. Both C4S mutant proteins and Filamin A are present in the lysosome. As a result, they may inhibit morphological differentiation. These results suggest a possible basis for the molecular and cellular pathological mechanisms that underlie infantile leukoencephalopathy.

## 2. Material and Methods

### 2.1. Primary and Secondary Antibodies

The following antibodies were purchased: mouse monoclonal antibody against a KDEL-containing peptide of the endoplasmic reticulum (ER)-resident glucose-regulated protein (GRP78) (Cat. No. M181-3; immunoblotting (IB), 1/10,000; and immunofluorescence (IF), 1/200), mouse monoclonal anti-actin (for the ß type; Cat. No. M177-3; IB, 1/40,000), mouse monoclonal anti-DDDDK antigen (also called FLAG antigen, Cat. No. M185-3; IB, 1/20,000), and mouse monoclonal anti-GFP (Cat. No. M048-3; and IB, 1/1000) from MBL (Aichi, Japan); mouse monoclonal anti-Golgi matrix protein of 130 kDa (GM130) (Cat. No. 610822; IB, 1/500; and IF, 1/200) from BD Biosciences (Franklin Lakes, NJ, USA); rabbit polyclonal anti-late endosomal Rab7 (Cat. No. 9367S; IF, 1/100) from Cell Signaling Technology (Danvers, MA, USA); goat polyclonal anti-lysosome-specific Catalase (Cat. No. AF3398; IF, 1/200) from Bio-Techne (Minneapolis, MN, USA); mouse monoclonal anti-lysosomal-associated membrane protein 1 (LAMP1) (Cat. No. ab25630; IB, 1/10,000; and IF, 1/100), rabbit monoclonal anti-Cathepsin D (Cat. No. ab75852; IF, 1/100), rabbit monoclonal anti-Rab9 (Cat. No. ab179815; IF, 1/100), rabbit monoclonal anti-Filamin A (Cat. No. ab 76289; immunoprecipitation (IP), 0.25 μg/500 μg of proteins; IB, 1/40,000; and IF, 1/100), mouse monoclonal anti-filamentous actin (F-actin, Cat. No. ab205; IF, 1/100), rabbit monoclonal anti-(pSer240/244) S6 protein/RPS6, which recognizes a phosphorylated state by p70S6K (Cat. No. ab215214; IB, 1/20,000), rabbit polyclonal anti-S6 protein/RPS6 (Cat. No. ab70227; IB, 1/250), rabbit monoclonal anti-(pThr37) 4E-BP1/eIF4E-BP1, which recognizes a phosphorylated state by mTOR kinase (Cat. No. ab75767; IB, 1/2500), rabbit monoclonal anti-4E-BP1 (Cat. No. ab32024; IB, 1/5000), and rabbit polyclonal anti-lysosome-specific transmembrane protein SLC38A9 (Cat. No. ab130398; IP, 1 μg/500 μg of proteins; and IB, 1/500) from Abcam (Cambridgeshire, UK); rabbit polyclonal anti-proteolipid protein 1 (PLP1) (Cat. No. HPA004128; IB, 1/500) from Atlas Antibodies (Bromma, Sweden); and rabbit polyclonal anti-C11ORF73 (Cat. No. 20524-1-AP; IF, 1/100) from Proteintech (Rosemont, IL, USA).

The following secondary antibodies were purchased: anti-rabbit or mouse IgG F(ab’) conjugated with horseradish peroxidase (Cat. Nos. 458 or 330; IB, 1/5000) from MBL; and anti-rabbit or mouse IgG (H+L) conjugated with Alexa Fluor 488 (Cat. Nos. A-11008 or A-11001; IF, 1/500) and anti-rabbit or mouse IgG (H+L) conjugated with Alexa Fluor 594 (Cat. Nos. A-11012 or A-11005; IF, 1/500) from Thermo Fisher Scientific (Waltham, MA, USA).

### 2.2. Plasmid Constructions

The plasmid encoding the human full-length *c11orf73* (GenBank Acc. No. NM_016401) gene was amplified from SuperScript III reverse transcriptase (Thermo Fisher Scientific)-mediated human brain cDNAs (human RNA originally from Nippon Gene Co. Ltd., Tokyo, Japan) using Gflex DNA polymerase (Takara Bio, Shiga, Japan), according to the manufacturer’s instructions. The amplified fragments were ligated with the GFP-expressing pEGFP-C1 vector (Takara Bio).

The Cys4-to-Ser (C4S; 11G-to-C in the nucleotide level) and Val54-to-Leu (V54L; 160G-to-C in the nucleotide level; OMIN ID 616881) mutations were produced from pEGFP-C1-C11ORF73 as the template using a site-directed mutagenesis kit (Takara Bio), in accordance with the manufacturer’s instructions. All DNA sequences were confirmed by sequencing (Fasmac, Kanagawa, Japan).

### 2.3. Cell culture, Differentiation, Transfection, and Isolation of Stable Clones

African green monkey kidney epithelial cell-like COS-7 and human glial T98G cells (Human Health Science Research Resource Bank, Osaka, Japan) were cultured on cell culture dishes (Greiner, Oberösterreich, Germany) in Dulbecco’s modified Eagle medium (DMEM) containing 10% heat-inactivated FBS and PenStrep (Thermo Fisher Scientific) in 5% CO_2_ at 37 °C.

Mouse brain oligodendroglial FBD-102b cells were cultured on cell culture dishes in DMEM/Nutrient Mixture F-12 containing 10% heat-inactivated FBS and PenStrep in 5% CO_2_ at 37 °C. To induce differentiation, FBD-102b cells were cultured on cell culture dishes (Greiner) with advanced TC polymer modification in culture medium without FBS for several days in 5% CO_2_ at 37 °C. Cells with multiple processes from the cell bodies and myelin web-like membrane structures were identified as differentiated [14]. Cells with a differentiated phenotype were considered to be those of cellular areas with a field of 25 square micrometers or more. FBD-102b cells were kindly provided by Dr. Y. Tomo-oka (Tokyo University of Science, Chiba, Japan).

Cells were transfected with the respective plasmids using a ScreenFect A or ScreenFect A Plus transfection kit (Fujifilm, Tokyo, Japan) according to the manufacturer’s instructions. The medium was replaced four hours after transfection and was generally used for experiments 48 h after transfection. Transfection efficiencies of COS-7 cells were approximately 75% as described in the manufacturer’s instructions. Transfection efficiencies of FBD-102b cells are 25 ± 5.0%, 27 ± 2.7%, and 26 ± 2.2% for the wild type, C4S, and V54L C11ORF73, respectively. Thus, COS-7 cells but not FBD-102b cells are suitable for biochemical experiments such as analyzing protein properties that need high level expression of proteins. To confirm the viability of COS-7 cells and FBD-102b cells under each experimental condition, we verified that attached trypan-blue-incorporating cells made up less than 5% of all cells in each culture [14].

To collect FBD-102b cells stably harboring the wild type or the mutant constructs of C11ORF73, cells were transfected with the respective plasmids in a 3.5-cm cell culture dish. Growth medium containing 0.1250 mg/mL G418 (Nacalai Tesque, Kyoto, Japan), was changed every two or three days. After approximately 14 days, G418-resistant colonies were collected and their phenotypes were compared with those of their parental or control cells.

### 2.4. Fluorescence Images

Cells on a coverslip were fixed with 4% paraformaldehyde or 100% cold methanol. They were blocked with Blocking One reagent (Nacalai Tesque) and incubated with a primary antibody and an Alexa Fluor-conjugated secondary antibody (Thermo Fisher Scientific). For F-actin staining, cells were incubated with Phalloidin-iFluor594 reagent (Cat. No. ab176757, Abcam; IF 1/1000). The coverslips on the slide glass were mounted with Vectashield reagent (Vector Laboratories, Burlingame, CA, USA). TIFF images were collected with a microscope system equipped with a laser-scanning Fluoview apparatus (FV1000D or FV1200, Olympus, Tokyo, Japan) using Fluoview software (Olympus). The resulting color images were measured with the line and region analyses using Image J software for line plots and merged percentages, respectively. Each image in the figures is the representative of three independent experimental results (*n* = 25 cells in total).

### 2.5. Polyacrylamide Gel Electrophoresis and Immunoblotting

Cells were lysed in lysis buffer A (50 mM HEPES-NaOH, pH 7.5, 150 mM NaCl, 20 mM MgCl_2_, 1 mM phenylmethane sulfonylfluoride, 1 μg/mL leupeptin, 1 mM EDTA, 1 mM Na_3_VO_4_, 10 mM NaF, and 0.5% NP-40) [15,16]. For non-denatured or denatured conditions, the supernatants were incubated with non-denaturing or denaturing sample buffer (Nacalai Tesque), respectively. The samples were separated on pre-made non-denatured (native) or denatured polyacrylamide gels (Nacalai Tesque). The electrophoretically separated proteins were transferred to polyvinylidene difluoride membranes (Merck Millipore, Darmstadt, Germany), blocked with Blocking One reagent, and immunoblotted using first primary antibodies, then horseradish peroxidase (HRP)-conjugated secondary antibodies. The bound antibodies were detected by X-ray film exposure using ImmunoStar Zeta reagent (Fujifilm). The films were captured as TIFF image files using a Canon LiDE 400 scanner (Canon, Tokyo, Japan) and its driver software (Canon). The band pixels were measured with the segment analysis using UN-SCAN-IT software. The pixel values of immunoreactive bands were reported as percentages and were compared with the control values. Each image in the figures is representative of three independent experimental results.

### 2.6. Immunoprecipitation for the Intended Protein or the Lysosome

Supernatants of the cell lysates in buffer A were used for immunoprecipitation of the purposed proteins [15,16]. The supernatants were mixed with protein G resin (GE Healthcare, Fairfield Easton, CT, USA) that had been absorbed with an antibody. The immunoprecipitates in supernatants of the cell lysate were denatured, subjected to polyacrylamide gel electrophoresis, and blotted onto polyvinylidene difluoride membranes for immunoblotting.

For immunoprecipitation of the lysosome, we used buffer B (50 mM HEPES-NaOH, pH 7.5, 150 mM NaCl, 5 mM MgCl_2_, 1 mM phenylmethane sulfonylfluoride, 1 μg/mL leupeptin, 1 mM EDTA, 1 mM Na_3_VO_4_, 10 mM NaF) and homogenized cell lysates with Potter-Elvehjem homogenizer. The homogenates were centrifuged at 150× *g* for 10 min using a tabletop centrifuge. The supernatants were gently mixed with an anti-SLC38A9 antibody-absorbed protein G resin [17]. The immunoprecipitates were denatured, subjected to polyacrylamide gel electrophoresis, and blotted onto membranes for immunoblotting.

### 2.7. Mass Spectrometry (MS)-Based Identification of Affinity-Precipitated C11ORF73-Binding Proteins

Human glial cell line T98G was infected with retroviruses harboring either the gene encoding C11ORF73 or its mutant (C4S or V54L) tagged with FLAG and hexa-histidine in tandem and puromycin-resistant gene. These experiments were virus-mediated transfections, since their efficiency almost reaches 100%. After puromycin selection, cells were harvested and lysed with RIPA Buffer (50 mM sodium phosphate, pH 7.4, 1 mM MgCl_2_, 150 mM NaCl, 1% Nonidet P-40, 0.8% deoxycholate, 100 mM Na_3_VO_4_, 10 mM NaF, and protease inhibitors; Fujifilm). Using cell lysates, protein complexes including C11ORF73 or one of its mutants were purified through sequential affinity chromatography including M2-conjugated (Sigma-Aldrich, St. Louis, MO, USA) and Ni-NTA agarose (Qiagen, Venlo, Netherlands). Purified fractions were concentrated according to the 20% TCA precipitation method and degraded with 1 U/mL Trypsin Gold (Promega, Madison, WI, USA) overnight. To prevent re-formation of the disulfide bond between degraded peptides, thiol groups were masked by treatment with iodoacetamide. Trypsin-degraded peptides were separated by L-column 2 ODS (CERI, Saitama, Japan) by gradient with acetonitrile: 0 to 60% in 0.1 M trifluoroacetic acid. The fractions were mixed with α-cyano-4-hydroxy cinnamic acid and spotted onto MTP Anchor Chip 384 (Bruker Daltonics, Billerica, MA, USA). Peptides were analyzed in an Autoflex speed analyzer (Bruker Daltonics) for MALDI-TOF/TOF analysis, and proteins were identified using a Mascot server (Matrix Science, Tokyo, Japan).

### 2.8. Statistical Analysis

Values are means ± SD from separate experiments. Intergroup comparisons were performed according to Student’s t test using Microsoft Excel (Redmond, WA, USA). A one-way analysis of variance (ANOVA) was followed by a Fisher’s protected least significant difference test as a post hoc comparison using AnalystSoft StatPlus software. Differences were considered significant when *p* < 0.05.

### 2.9. Ethics Statement

Gene recombination techniques were performed in vitro and in vivo in accordance with a protocol approved by both the Tokyo University of Pharmacy and Life Sciences Gene and Animal Care Committee and the Japanese National Research Institute for Child Health and Development Gene and Animal Care Committee (L18-04 (data of approval, 4/1/2018), L19-04 (data of approval, 4/1/2019), and L20-04 (data of approval, 4/1/2000)).

## 3. Results

### 3.1. The C4S Mutant Proteins of C11ORF73 are Localized in the Lysosome and Form Oligomers

**Figure 1:**
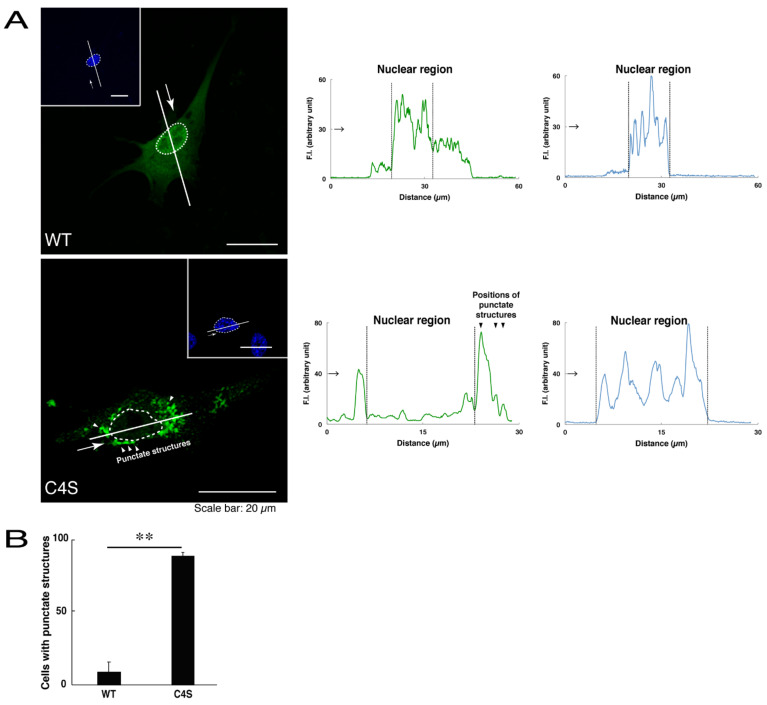
The Cys4-to-Ser (C4S) mutant proteins of C11ORF73 accumulate into punctate structures in cells. (**A**) FBD-102b cells (oligodendroglial cell lines), which are surrounded by dotted lines, were transfected with a plasmid encoding the wild type (WT) or the C4S mutant construct of GFP-tagged C11ORF73 (green) and stained with DAPI (blue). A scan plot was performed along the white line in the direction of the arrow in the image. Graphs showing the fluorescence intensities (F.I., in an arbitrary unit) along the white lines can be seen in the right panels. Some punctate structures (indicated by arrowheads) and nuclear regions (surrounded by dotted lines) are also shown in the images and graphs. (**B**) Percentages of cells with punctate structures are statistically shown (**, *p* < 0.01 of Student’s *t*-test; n = 25 cells).

We first investigated whether the infantile leukoencephalopathy-associated C4S mutant proteins of C11ORF73 are localized, like the wild type C11ORF73 proteins, in the cytoplasmic and nuclear regions. We transfected the plasmid encoding the wild type construct or the C4S mutant construct tagged with green fluorescent protein (GFP) into oligodendroglial FBD-102b cells. While transfected wild type C11ORF73 proteins were indeed localized in the cytoplasmic and nuclear regions [11,12], approximately 80% of the mutant proteins were observed in the punctate structures of the cytoplasmic regions (Figure 1A,B). Similarly, endogenous C11ORF73 proteins, which were recognized by an anti-C11ORF73 antibody, were localized in the cytoplasmic and nuclear regions, consistent with subcellular localizations of transfected C11ORF73 proteins (Figure S1). In contrast, transfected exogenous mutant proteins, which were recognized by an anti-C11ORF73 antibody, were indeed observed in the punctate structures (Figure S2).

**Figure 2:**
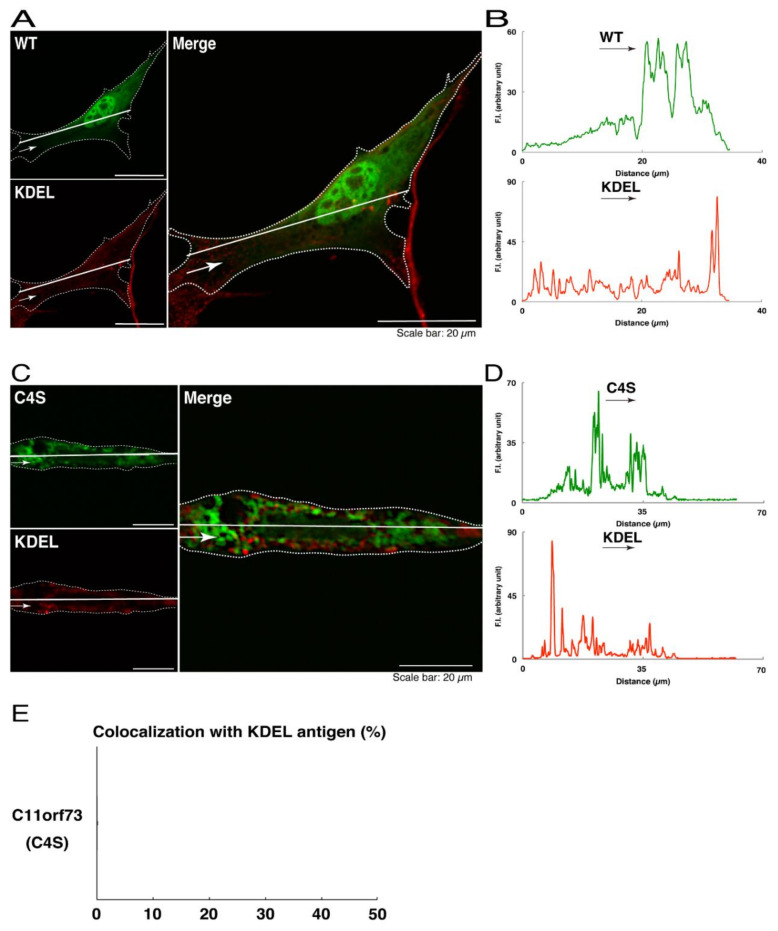
The C4S mutant proteins of C11ORF73 are not colocalized with the endoplasmic reticulum (ER) marker. (**A**) FBD-102b cells, which are surrounded by dotted lines, were transfected with a plasmid encoding the wild type (WT) GFP-tagged C11ORF73 (green) and stained with the ER marker KDEL antigen (red). A scan plot was performed along the white line in the direction of the arrow in the image. (**B**) Graphs showing the fluorescence intensities (F.I., in an arbitrary unit) along the white lines can be seen in the panels. (**C**) Cells were transfected with a plasmid encoding the C4S mutant construct of GFP-tagged C11ORF73 (green) and stained with the ER marker (red). A scan plot was performed along the white line in the direction of the arrow in the image. (**D**) Graphs showing the fluorescence intensities (F.I., in an arbitrary unit) along the white lines can be seen in the panels. (**E**) Merged percentages of mutant proteins with organelles (percentages of yellow-colored pixels/green-colored pixels) are shown in the graph (*n* = 3 independent experiments).

**Figure 3:**
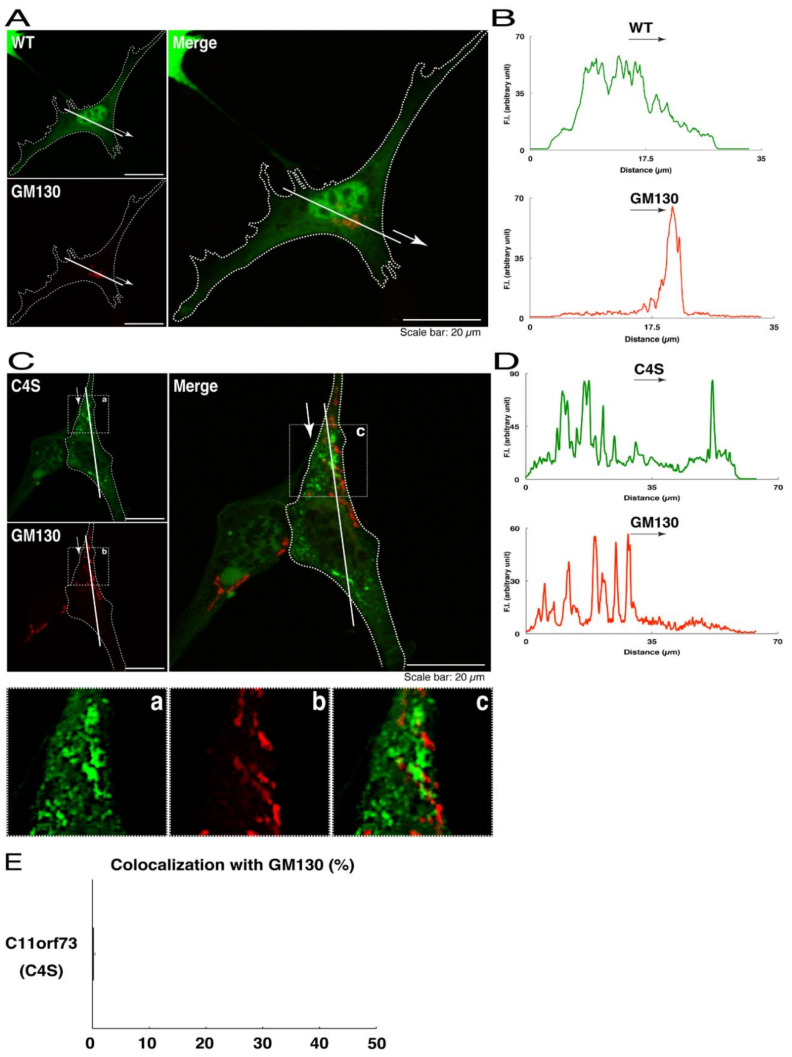
The C4S mutant proteins of C11ORF73 are not colocalized with the Golgi body marker. (**A**) FBD-102b cells, which are surrounded by dotted lines, were transfected with a plasmid encoding the wild type (WT) GFP-tagged C11ORF73 (green) and stained with the Golgi body marker GM130 (red). A scan plot was performed along the white line in the direction of the arrow in the image. (**B**) Graphs showing the fluorescence intensities (F.I., in an arbitrary unit) along the white lines can be seen in the panels. (**C**) Cells were transfected with a plasmid encoding the C4S mutant construct of GFP-tagged C11ORF73 (green) and stained with the Golgi body marker (red). Regions (**a**–**c**) within white squares are magnified in the below panels. A scan plot was performed along the white line in the direction of the arrow in the image. (**D**) Graphs showing the fluorescence intensities (F.I., in an arbitrary unit) along the white lines can be seen in the panels. (**E**) Merged percentages of mutant proteins with organelles (percentages of yellow-colored pixels/green-colored pixels) are shown in the graph (*n* = 3 independent experiments).

**Figure 4:**
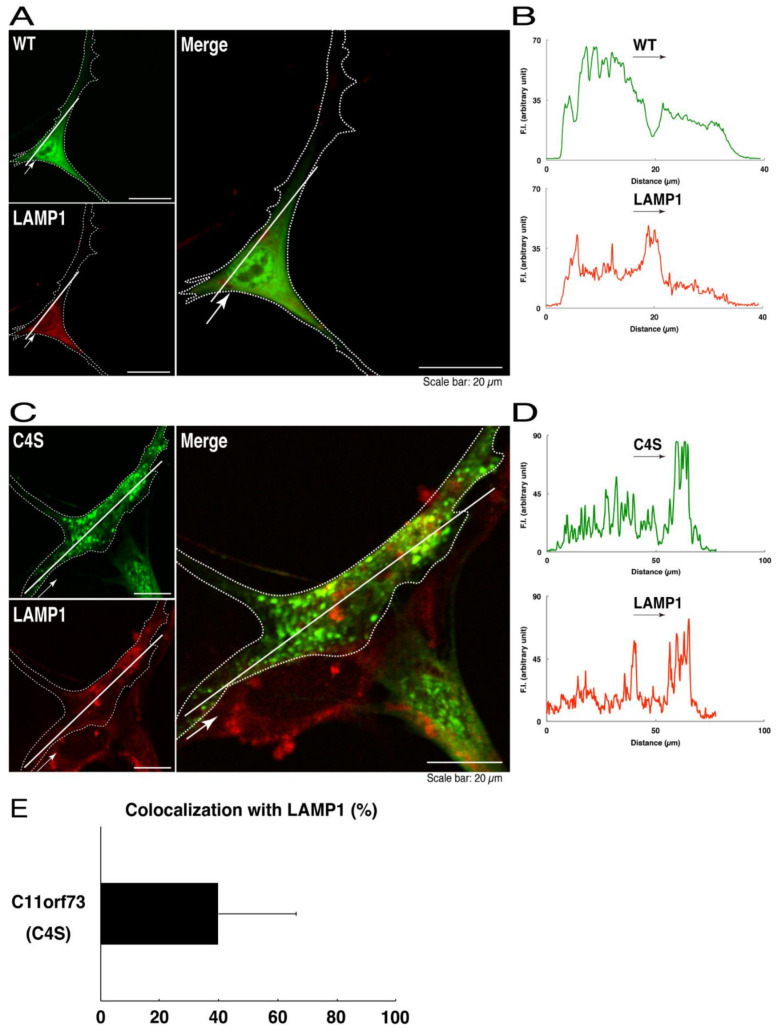
The C4S mutant proteins of C11ORF73 are colocalized with the lysosome marker. (**A**) FBD-102b cells, which are surrounded by dotted lines, were transfected with a plasmid encoding the wild type (WT) GFP-tagged C11ORF73 (green) and stained with the lysosome marker LAMP1 (red). A scan plot was performed along the white line in the direction of the arrow in the image. (**B**) Graphs showing the fluorescence intensities (F.I., in an arbitrary unit) along the white lines can be seen in the panels. (**C**) Cells were transfected with a plasmid encoding the C4S mutant construct of GFP-tagged C11ORF73 (green) and stained with the lysosome marker (red). A scan plot was performed along the white line in the direction of the arrow in the image. (**D**) Graphs showing the fluorescence intensities (F.I., in an arbitrary unit) along the white lines can be seen in the panels. (**E**) Merged percentages of mutant proteins with organelles (percentages of yellow-colored pixels/green-colored pixels) are shown in the graph (*n* = 3 independent experiments).

We thus tried to identify the organelle in which these punctate structures were located. We stained transfected cells with antibodies against the endoplasmic reticulum (ER) marker KDEL antigen, the Golgi body marker GM130, and the lysosome marker lysosomal-associated membrane protein 1 (LAMP1). The secondary antibody used in the immunofluorescence study failed to have the ability to stain the cells (Figure S3). The wild type and mutant proteins were not colocalized with either KDEL antigen or GM130 (Figure 2A–D; Figure 3A–D). As for the lysosome marker, the mutant proteins displayed colocalization with a commonly used lysosome-specific antibody against LAMP1, while the wild type proteins did not (Figure 4A–D). Similar results were observed with the anti-lysosome-specific Cathepsin D antibody (Figure S4). In addition, we stained transfected cells with antibodies against Rab7 and Rab9, since an anti-LAMP1 antibody likely has the ability to stain Rab7 or Rab9 vesicles [18]. Neither the anti-Rab7 antibody nor the anti-Rab9 antibody yielded a significant amount of staining in the wild type C11ORF73 proteins and the mutant proteins (Figures S5 and S6). These results suggest that the C4S mutation of C11ORF73 causes C11ORF73 to be localized in the lysosome.

To obtain more data for comparison with this finding, we also examined the effects of the HLD13-associated V54L mutation on the cellular localization of C11ORF73 proteins. We stained transfected cells expressing the V54L mutant proteins with antibodies against KDEL antigen, GM130, and LAMP1. Like the C4S mutant proteins, the V54L mutant proteins were arranged into punctate structures (Figure S7). In addition, these mutant proteins, like the C4S mutant proteins and the wild type proteins, were not colocalized with either KDEL antigen or GM130 (Figures S8 and S9). Unlike the C4S mutant proteins, however, the V54L proteins were not colocalized with LAMP1 (Figure S10), suggesting that the V54L mutation of C11ORF73 does not necessarily cause its localization into the lysosome.

**Figure 5:**
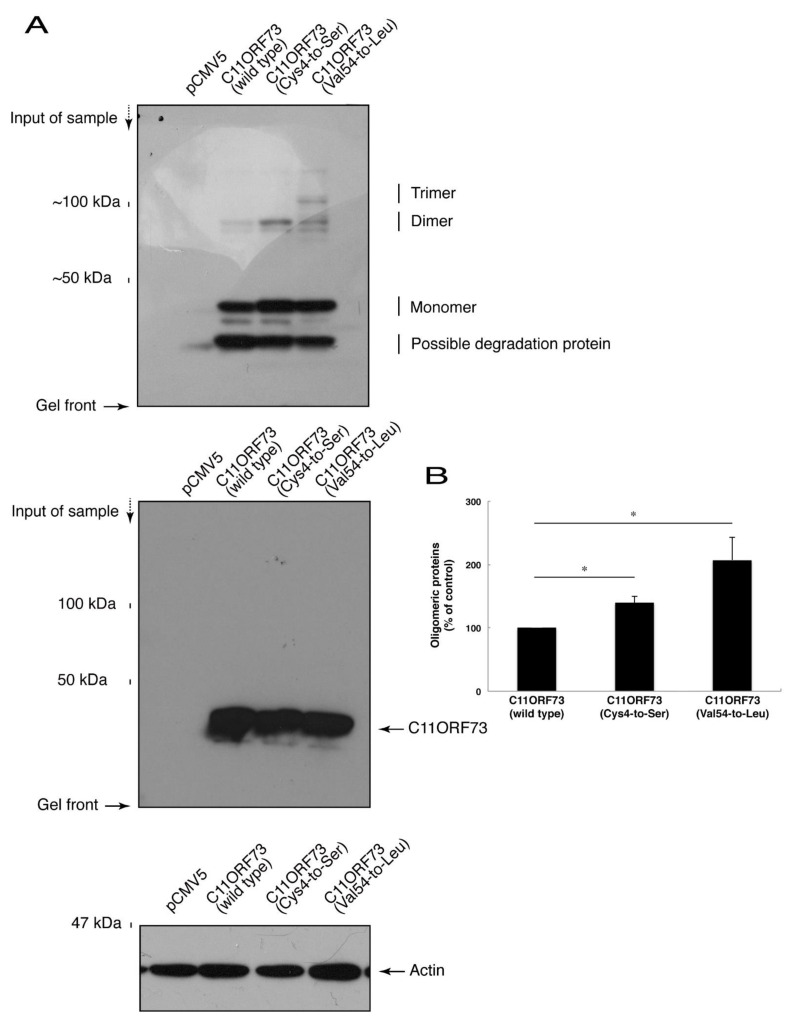
The C4S as well as the V54L mutant proteins of C11ORF73 exhibit oligomeric structures in non-denaturing polyacrylamide gel electrophoresis. (**A**) The lysates of COS-7 cells were transfected with empty vectors or with a plasmid encoding wild type, Cys4-to-Ser (C4S), or Val54-to-Leu (V54L) C11ORF73, and then were subjected to non-denaturing polyacrylamide gel electrophoresis and immunoblotted with an anti-GFP antibody. Positions corresponding to the molecular weights of the C11ORF73 monomer, dimer, and trimer are also shown, and the oligomeric proteins of molecules with more than three units can be seen in the V54L mutant proteins. The lysates were also subjected to denaturing polyacrylamide gel electrophoresis and immunoblotted with an anti-actin or anti-GFP antibody. Immunoreactive bands below monomer could be degradative proteins of C11ORF73. (**B**) Immune-reactive band intensities in a range containing oligomeric proteins were quantitatively compared to those of wild-type proteins. (*, *p* < 0.05 of one-way analysis of variance (ANOVA) with post-hoc Fisher’s test; *n* = 3 blots).

Since the C4S mutation of C11ORF73 proteins leads to its localization in the lysosome, we explored whether this mutation also causes protein aggregation. We subjected cell lysates expressing the wild type or mutant proteins to non-denaturing polyacrylamide gel electrophoresis. The mobilizing position of the wild type proteins primarily corresponded to the monomeric position. In addition, the mobilizing positions of the C4S and V54L mutant proteins involved immunoreactive band positions corresponding to the dimeric and trimeric/polymeric positions, respectively (Figure 5A,B), revealing that the C4S as well as the V54L mutation of C11ORF73 can cause protein aggregation.

### 3.2. Cells Harboring the C4S Mutant Constructs of C11ORF73 Exhibit Inhibited FBD-102b Cell Morphological Differentiation

**Figure 6:**
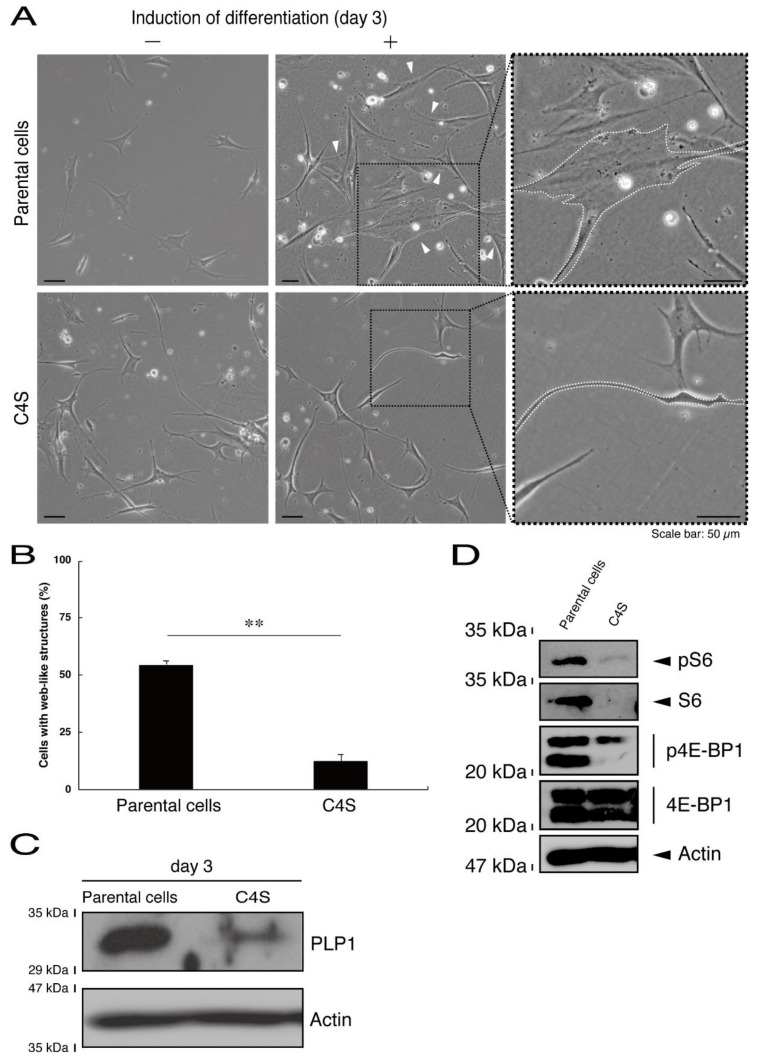
Cells harboring the C4S mutant constructs fail to exhibit differentiated phenotypes. (**A**) FBD-102b cells stably harboring the C4S mutant constructs or their parental cells were allowed to differentiate for three days. Arrowheads indicate cells with web-like structures as an example of a differentiated phenotype. Some cells are surrounded by white dots. Square fields with dotted lines in the center panels are magnified in the right panels. (**B**) Cells with web-like structures are statistically shown (**, *p* < 0.01 of one-way ANOVA with post-hoc Fisher’s test; *n* = 3 fields). (**C**) The lysates of the respective cells were immunoblotted with an antibody against proteolipid protein 1 (PLP1) or control actin. (**D**) The lysates were also immunoblotted with an antibody against phosphorylated S6 (pS6), S6, phosphorylated 4E-BP1 (p4E-BP1), 4E-binding protein 1 (4E-BP1), or actin.

**Figure 7:**
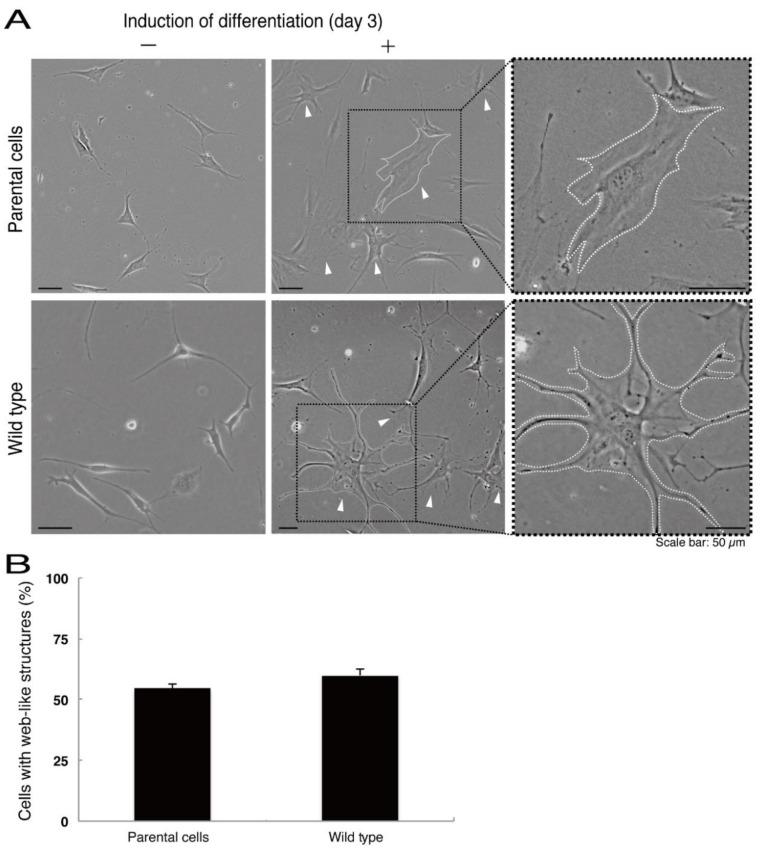
Cells harboring the wild type constructs exhibit the same differentiated phenotypes as their parental cells. (**A**) FBD-102b cells stably harboring the wild type constructs or their parental cells were allowed to differentiate for three days. Arrowheads indicate cells with web-like structures as an example of a differentiated phenotype. Some cells are surrounded by white dots. Square fields with dotted lines in the center panels are magnified in the right panels. (**B**) Cells with web-like structures are statistically shown (*n* = 3 fields).

Next, we asked whether the C4S mutation of C11ORF73 proteins affects oligodendrocyte differentiation. We obtained FBD-102b cells stably harboring the C4S mutant constructs by means of antibiotic reagent-resistance. While parental FBD-102b cells had the ability to extend long processes from their cell bodies and, as a result, the ability to form myelin web-like structures following the induction of differentiation [14], these abilities were suppressed in cells harboring the C4S mutant constructs (Figure 6A,B). Importantly, cells harboring the C4S mutant constructs exhibited low expression levels of PLP1, which is a typical marker of oligodendrocyte myelin (Figure 6C). We further investigated the effects of the C4S mutation on signaling through mammalian target of Rapamycin (mTOR). Phosphorylation of ribosomal S6 and eukaryotic translation initiation factor 4E-binding protein 1 (4E-BP1) proteins as the outputs of signaling through mTOR are known to be associated with oligodendrocyte differentiation and myelination [19,20]. Immunoblotting with an anti-phosphorylated S6 or 4E-BP1 antibody revealed decreased phosphorylation levels in cells harboring the C4S mutant constructs (Figure 6D). When S6 or 4E-BP1 proteins are phosphorylated, the expression levels of S6 proteins are also known to be decreased [21], yet the expression levels of S6 or 4E-BP1 proteins were comparable in cells expressing the wild type and C4S mutant constructs. In addition, cells harboring the wild type constructs were as able to exhibit differentiated phenotypes as parental cells were (Figure 7A,B).

### 3.3. The C4S Mutant Proteins of C11ORF73 Specifically Interact with Filamin A and are Present in the Lysosome

**Figure 8:**
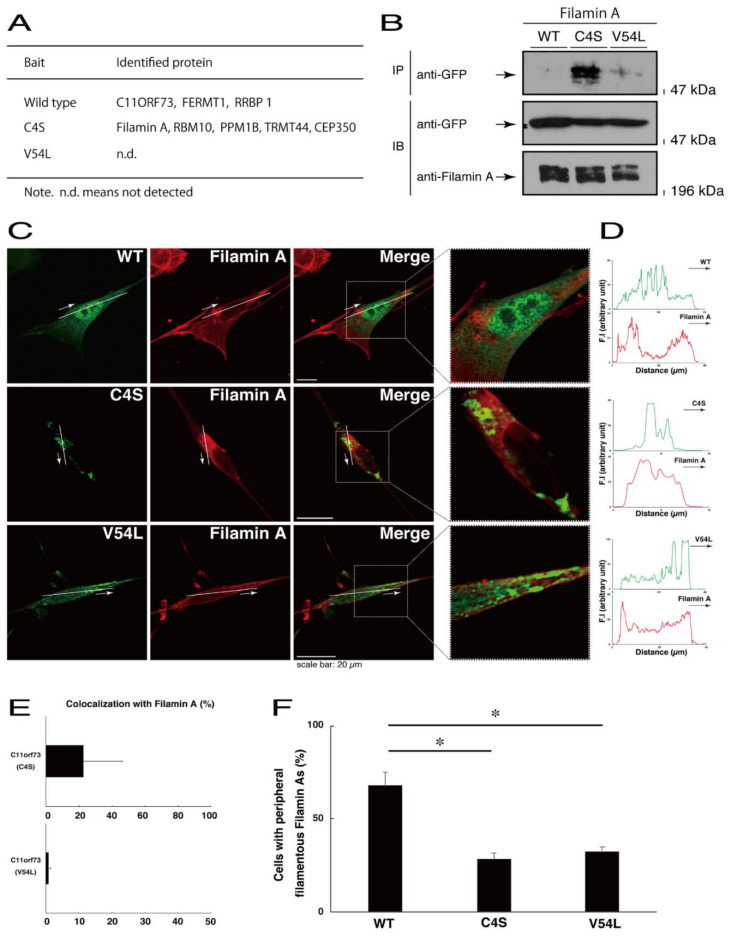
Filamin A specifically interacts with the C4S mutant proteins which inhibit the formation of filamentous Filamin A structures at cell peripheral regions. (**A**) Proteins interacting with the wild type, C4S, or V54L mutant proteins (bait) were identified by protein-tag affinity-precipitation and MS analysis. (**B**) COS-7 cells were transfected with a plasmid encoding one of the two C11ORF73 mutant constructs or the wild type (WT). The lysates were immunoprecipitated with an anti-Filamin A antibody in preparation for immunoblotting with an anti-GFP (for C11ORF73 proteins) antibody. Total C11ORF73 and Filamin A proteins are shown. (**C**,**D**) FBD-102b cells were transfected with plasmids encoding one of the C11ORF73 constructs (green) and stained with an anti-Filamin A antibody (red). Regions within white squares in merged images are magnified in the right panels. A scan plot was performed along the white line in the direction of the arrow in the image. (**E**) Merged percentages of mutant proteins with organelles (percentages of yellow-colored pixels/green-colored pixels) are shown in the graph (*n* = 3 independent experiments). (**F**) Cells with peripheral filamentous structures of Filamin A proteins are statistically shown (*, *p* < 0.05 of one-way ANOVA with post-hoc Fisher’s test; *n* = 3 fields).

**Figure 9:**
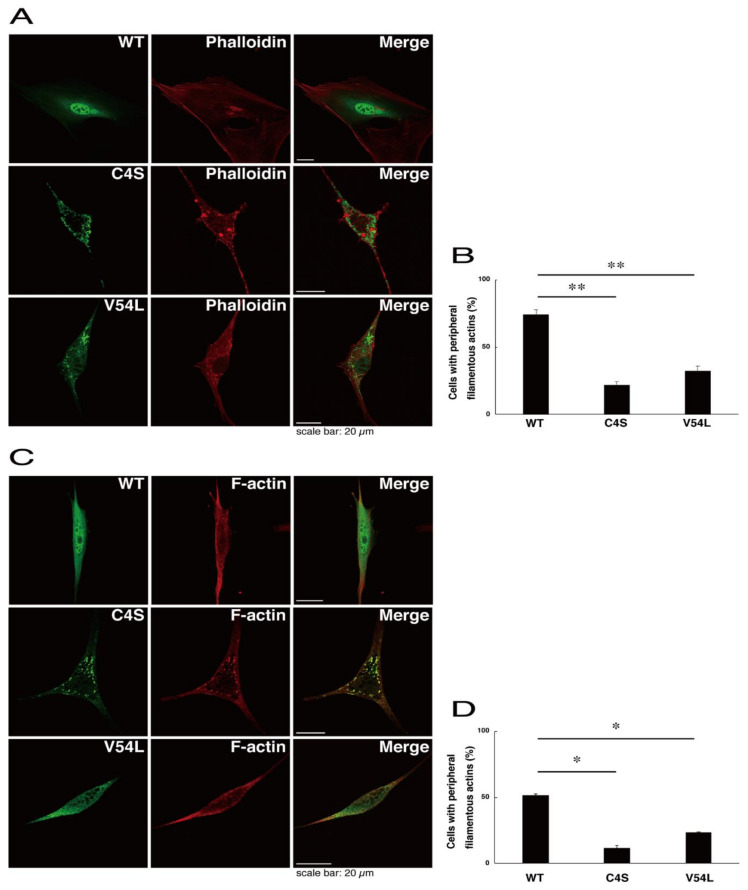
Formation of filamentous actin structures at cell peripheral regions is inhibited in cells expressing C4S mutant proteins. (**A**,**B**) FBD-102b cells were transfected with plasmids encoding one of the C11ORF73 mutant constructs or the wild type (WT) (green) and stained with Phalloidin (red). Cells with peripheral filamentous structures of actin proteins are statistically shown (**, *p* < 0.01 of one-way ANOVA with post-hoc Fisher’s test; *n* = 3 fields). (**C**,**D**) Cells were transfected with plasmids encoding one of the C11ORF73 constructs (green) and stained with an anti-F-actin antibody (red). Cells with peripheral filamentous structures of actin proteins are statistically shown (*, *p* < 0.05 of one-way ANOVA with post-hoc Fisher’s test; *n* = 3 fields).

To explore potential mechanisms whereby the C4S mutant proteins of C11ORF73 could decrease differentiated phenotypes, we set out to identify proteins that interact specifically with the C4S mutant proteins (Figure S11 for the wild type proteins; Figure S12 for the C4S mutant proteins; and Figure S13 for the V54L mutant proteins). Mass spectrometry analysis enabled us to identify their specific interactive proteins (Figure 8A). We focused on the actin-binding Filamin A [13] as the C4S mutant-specific interactive protein, since the formation and rearrangement of the actin cytoskeletal networks are essential for oligodendrocyte differentiation and myelination [22]. We first performed an immunoprecipitation of the C4S mutant proteins with endogenous Filamin A proteins in COS-7 cells. The C4S mutant proteins formed an immune-complex with Filamin A, whereas neither the V54L mutant proteins nor the wild type proteins did so (Figure 8B). Furthermore, immunofluorescence studies showed colocalization of the C4S mutant proteins, but not of the V54L mutant or wild type proteins, with endogenous Filamin A in punctate structures in FBD-102b cells (Figure 8C–E). Cells expressing wild type C11ORF73 proteins were surrounded with filamentous Filamin A proteins at the cell peripheral regions; the expression of C4S or V54L mutant proteins, in contrast, decreased the numbers of these filamentous proteins (Figure 8F). In addition, we stained cells expressing the wild type, C4S mutant, or V54L mutant proteins with Phalloidin, which specifically binds to filamentous actins (also called F-actins). Consistent with the results we obtained through staining with Phalloidin, the C4S or V54L mutant proteins decreased filamentous actins at cell peripheral regions (Figure 9A,B). Similar results were obtained through staining with an anti-F-actin antibody (Figure 9C,D). Together, these results suggest that the C4S mutant proteins specifically interact with Filamin A, probably to inhibit the formation or rearrangement of actin cytoskeletal networks and in turn the morphological differentiation of FBD-102b cells.

**Figure 10:**
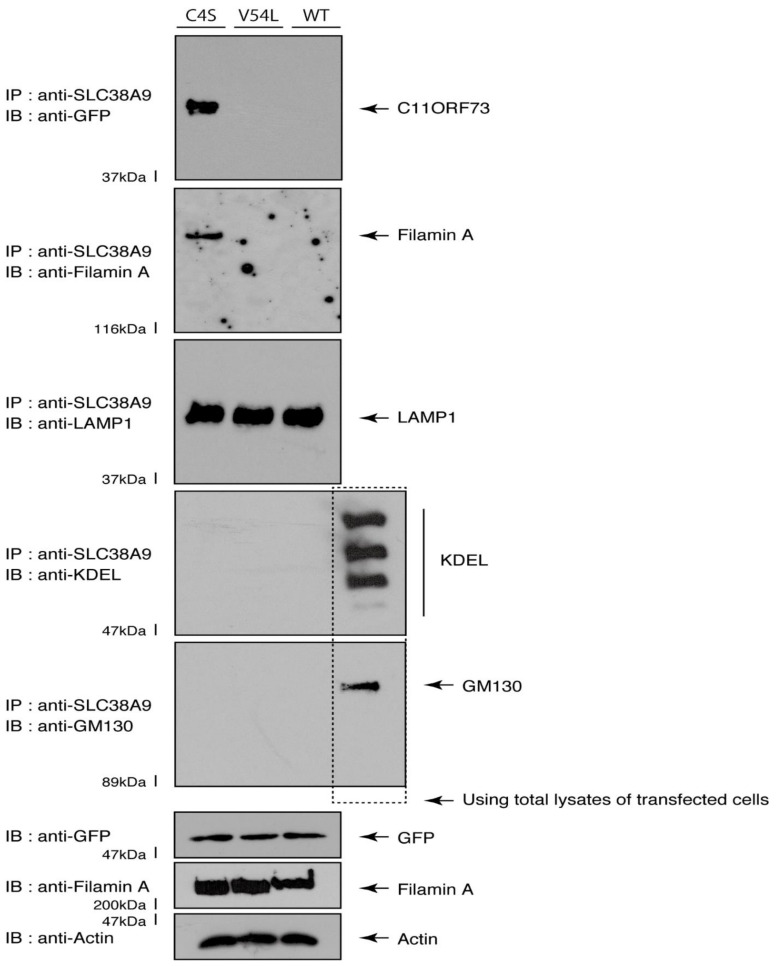
The C4S mutant proteins and Filamin A are present in the lysosome fraction. COS-7 cells were transfected with a plasmid encoding the wild type (WT), C4S, or V54L mutant construct. The lysates obtained from cells destroyed with isotonic solution were immunoprecipitated with an anti-SLC38A9 antibody in preparation for immunoblotting with an antibody against Filamin A or green fluorescent protein (GFP) (for C11ORF73 proteins). Immunoprecipitates were also immunoblotted with antibodies against KDEL antigen, Golgi matrix protein of 130 kDa (GM130), and lysosomal-associated membrane protein 1 (LAMP1), whose immunoreactive bands indicated that control proteins were present in the lysates of total cells. Total Filamin A, C11ORF73, and actin proteins can be seen in the lower three blots.

Given that the C4S mutant proteins are colocalized with Filamin A, we next asked whether both proteins are present in the lysosome. We transfected the plasmids encoding the wild type, C4S, or V54L proteins into FBD-102b cells and used their lysates for immunoprecipitation with an antibody against solute carrier family 38 member 9 (SLC38A9), a transmembrane protein in the lysosome [17]. The lysosome recovered with this anti-SLC38A9 antibody contained C4S mutant proteins but not V54L or wild type proteins (Figure 10). Filamin A proteins were detected only in the lysosomes of cells expressing the C4S mutant proteins (Figure 10). Notably, an immuno-complex with an anti-SLC38A9 antibody contained LAMP1 but did not contain KDEL antigen or GM130. Protein expression levels in transfected cells were comparable among the various proteins. It is thus suggested that Filamin A and the C4S mutant proteins are present in the lysosome fraction.

## 4. Discussion

C11ORF73 mediates the heat-shock-induced nuclear uptake of 70 kDa heat-shock proteins through interactions with nucleoporins. In response to heat-shock, C11ORF73 forms a complex with ATP-bound 70 kDa heat-shock proteins and supports the transport of heat-shock proteins into the nucleus [11]. Recent studies have illustrated that two types of C11ORF73 mutations, C4S and V54L, are responsible for the oligodendrocyte myelin-related disorders [10,12]. Although the C4S mutation is known to cause infantile leukoencephalopathy, to date, it has remained unclear how this mutation induces its pathological effects on oligodendrocytes at the molecular and cellular levels. Here, we show that the C4S mutant proteins aggregate and are abundantly localized in the lysosome, where they downregulate lysosomal signaling through S6 and 4E-BP1 phosphorylation. C4S mutant proteins, but not wild type proteins, specifically interact with Filamin A. Filamin A, which is also detected in the lysosome fraction, plays a key role in mediating the formation of a cytoplasmic actin network among actin filaments or between actin filaments and other molecules, and also contributes to cell morphological maintenance and change [22]. While cells harboring the wild type constructs normally exhibit differentiated phenotypes, cells harboring the C4S mutant ones fail to do so. These findings suggest that the C4S mutation can lead to defective morphological differentiation in oligodendrocytes, possibly by generating a de novo interactive ability with Filamin A and accumulating Filamin A in the lysosome.

Some nuclear proteins related to myelin-related disorders have been identified previously. The mutations of the genes encoding the RNA polymerase III subunits POLR3A and POLR3B are responsible for HLD7 and HLD8, for example [23]. RNA polymerase III functions as transcripting noncoding RNA and sometimes as doing tRNA. HLD7 is an autosomal recessive disorder characterized by childhood onset of progressive motor decline. The features involve spasticity, ataxia, and tremor. Other features may include hypodontia or oligodontia and hypogonadotropic hypogonadism [23]. HLD8 is an autosomal recessive disorder characterized by cerebellar ataxia and intellectual disabilities with oligodontia or hypogonadotropic hypogonadism [23]. It is likely that loss of function of RNA polymerase III activities causes HLD7 and HLD8 [23], but it remains unclear whether the observed disease features are actually due to downregulation of the transcription of noncoding RNAs. Alternatively, it is thought that mutations of RNA polymerase III subunit A (POLR3A) or RNA polymerase III subunit B (POLR3B) may induce aggregation of POLR3A and POLR3B proteins, leading to proteopathies (also called proteinopathies) associated with gain of function in POLR3A or POLR3B mutants. Indeed, the infantile leukoencephalopathy-associated C4S mutation of nuclear and cytoplasmic C11ORF73 also causes C11ORF73 protein aggregation, but it has remained unclear to date whether this protein aggregation was directly related to the leukoencephalopathy phenotypes observed in vivo.

The C4S mutant proteins were accumulated in the lysosome, since they might not be degraded; in contrast, the wild type proteins were not present in the lysosome. Alternatively, disease-associated mutation might change properties of organelle localization of proteins. In either case, evidence shows that signaling through mTOR is involved in the regulation of lysosome function and vice versa [24]. mTOR is present in two structurally distinct forms: mTOR complex 1 (mTORC1) and mTOR complex 2 (mTORC2). mTORC1 and mTORC2 specifically control distinct groups of the downstream effectors. mTORC1 links energy metabolism and amino acid balance to cell growth and proliferation. It is also involved in the regulation of cell volumes by molecular events such as protein synthesis including S6 and 4E-BP1 phosphorylation and in the utilization of energy stores. For example, when amino acid levels are high, mTORC1 is recruited to the lysosomal surface, where it is mediated by the Ras family small GTPase Rheb [24]. The translocation of mTOR to the lysosome participates in protein synthesis through S6 and 4E-BP1 phosphorylation. These effectors are associated with maintaining the functional lysosome, which can lead to the determination of cell fate through a possible regulation linking to transcription factor EB (TFEB) [25]. In this study, cells harboring wild type C11ORF73 constructs have the ability to promote the lysosome-related S6 and 4E-BP1 phosphorylation, whereas cells harboring C4S mutant proteins lack this ability. Furthermore, mTOR signaling through S6 and 4E-BP1 phosphorylation is important for oligodendrocyte differentiation and myelination [19,20]. Thus, it is conceivable that a decrease in phosphorylation level may be associated with inhibitory differentiation in FBD-102b cells.

We found that the C4S mutation of C11ORF73 gives C11ORF73 proteins the capacity for de novo interaction with Filamin A. This interaction inhibits F-actin formation in cells, perhaps because mutated aggregated C11ORF73 proteins cause Filamin A to accumulate in the lysosome. Cycles of F-actin organization and disorganization are necessary for the cell morphological changes underlying oligodendrocyte differentiation and myelination [22]. Filamin A is a 280 kDa actin-binding protein that binds to cell adhesion molecules and receptors and regulates actin cytoskeletal reorganization [13]. Filamin A is one of the central molecules linking upstream signaling to actin cytoskeletal changes [13]. In fact, mutations in Filamin A are responsible for a variety of genetic diseases related to cell morphological changes. These diseases are likely caused by loss-of-function of Filamin A. It is possible that, in infantile leukoencephalopathy tissues and cells, the C4S mutant proteins’ interaction with Filamin A inhibits F-actin formation and thereby inhibits cell morphological changes.

## 5. Conclusions

The present study is the first to report that the infantile leukoencephalopathy-associated C4S mutant proteins of C11ORF73 are localized in the lysosome where they aggregate and downregulate lysosome-related phosphorylating signals. We also report that this mutation gives C11ORF73 proteins the capacity for de novo interaction with Filamin A. Filamin A and C11ORF73 proteins are concomitantly detected in the lysosome fraction. Cells harboring the C4S mutant constructs do not exhibit differentiated phenotypes, whereas cells harboring wild type constructs do. Further studies will advance our understanding of the detailed mechanism by which the C4S mutant proteins are localized in the lysosome and downregulate lysosomal signaling. Signaling around the lysosome is known to be essential for oligodendrocyte differentiation and myelination, but it remains unclear how this signaling is associated with differentiation and myelination. Additionally, further analyses of the interaction between mutated C11ORF73 proteins and Filamin A may allow us to understand whether and how this interaction is actually responsible for the molecular and cellular pathological phenotypes associated with infantile leukoencephalopathy. Studies in this field may lead to the development of a drug-target-specific medicine for the treatment of infantile leukoencephalopathy and other possible C11ORF73-related diseases.

## Data Availability

Not applicable.

## Supplementary Materials

The following are available online at https://www.mdpi.com/2305-6320/8/2/9/s1, Figure S1. GFP-tagged C11ORF73 proteins exhibit similar localization to endogenous C11ORF73 ones. Figure S2. An anti-C11ORF73 antibody-stained C11ORF73 mutant proteins exhibit similar localization to an anti-GFP antibody-stained ones. Figure S3. Secondary antibodies have no abilities to stain cells. Figure S4. The C4S mutant proteins of C11ORF73 are colocalized with the Cathepsin D marker. Figure S5. The C4S mutant proteins of C11ORF73 are not colocalized with Rab7. Figure S6. The C4S mutant proteins of C11ORF73 are not colocalized with Rab9. Figure S7. The V54L mutant proteins of C11ORF73 accumulate in punctate structures in cells. Figure S8. The V54L mutant proteins of C11ORF73 are colocalized with the KDEL antigen. Figure S9. The V54L mutant proteins of C11ORF73 are not colocalized with GM130. Figure S10. The V54L mutant proteins of C11ORF73 are not colocalized with LAMP1. Figure S11. List of the proteins interacting with wild type C11ORF73. Figure S12. List of the proteins interacting with C4S C11ORF73. Figure S13. List of the proteins interacting with V54L C11ORF73.

## Abbreviations

ANOVA, analysis of variance; CNS, central nervous system; C4S, Cys4-to-Ser; DMEM, Dulbecco’s modified Eagle medium; ER, endoplasmic reticulum; F-actin, filamentous actin; GFP, green fluorescent protein; GM130, Golgi matrix protein of 130 kDa; GRP, glucose-regulated protein; HLD, hypomyelinating leukodystrophy; HRP, horseradish peroxidase; LAMP1, lysosomal-associated membrane protein 1; mTOR, mammalian target of Rapamycin; PLP1, proteolipid protein 1; PMD, Pelizaeus-Merzbacher disease; PNS, peripheral nervous system; POLR3A, RNA polymerase III subunit A; POLR3B, RNA polymerase III subunit B; pS6, phosphorylated ribosomal protein S6; p4E-BP1, phosphorylated eukaryotic translation initiation factor 4E-binding protein 1; Rheb, Ras homolog enriched in brain; SLC38A9, solute carrier family 38 member 9; TFEB, transcription factor EB; and V54L, Val54-to-Leu.
