# Supplementary material for: The Infantile Leukoencephalopathy-Associated Mutation of C11ORF73/HIKESHI Proteins Generates De Novo Interactive Activity with Filamin A, Inhibiting Oligodendroglial Cell Morphological Differentiation
Source: Medicines (Basel). 2021 Feb 1;8(2):9. doi: 10.3390/medicines8020009 (PMC7912763; doi:10.3390/medicines8020009)
Supplement: Supplementary file 1 [file medicines-08-00009-s001.zip › Figure S11.pdf]

BRUKER

Project Info

**Name:**	200115-hikeshi-wt	**Date:**	Jan 15, 2020

Sample Info & Protocols

**Name:**	200115-hikeshi-wt
**Date:**	Jan 15, 2020

Search Result Info

**Search Result:**	LC-MALDI_OtherMammals_Mascot_2020-01-15 19:00:42		
**Location:**	/200115-hikeshi-wt/200115-hikeshi-wt/200115-hikeshi-wt		
**Search Method:**	LC-MALDI_OtherMammals	**Version:**	
**Search Engine(s):**	Mascot, 2.6.2	**Ident. Compound(s):**	6/300
**Database(s):**	SwissProt, SwissProt_2019_07.fasta		
**Note:**			

Results

Protein 1: Protein Hikeshi OS=Homo sapiens OX=9606 GN=HIKESHI PE=1 SV=2

**Accession:**	HIKES_HUMAN	**Score:**	207.4
**Database:**	SwissProt	**Seq. Coverage [%]:**	16.2
**MW [kDa] / pI:**	21.6 / 5.3	**No. of Peptides:**	3

10	20	30	40	50	60	70	80	90	100
MFGCLVAGRL	VQTAAQQVAE	DKFVFDLPDY	ESINHVVVFM	LGTIPFPEGM	GGSVYFSYPD	SNGMPVWQLL	GFVTNGKPSA	IFKISGLKSG	EGSQHPFGAM
**110**	**120**	**130**	**140**	**150**	**160**	**170**	**180**	**190**	**200**
NIVRTPSVAQ	IGISVELLDS	MAQQTPVGNA	AVSSVDSFTQ	FTQKMLDNFY	NFASSFAVSQ	AQMTPSPSEM	FIPANVVLKW	YENFQRRLAQ	NPLFWKT

MS/MS Peptide Matches

Cmpd.	m/z meas.	z	Δ m/z [ppm]	Δ m/z [Da]	Rt [min]	Score	No. of Cmpds.	Site [%]	P	Sequence	Modification	Range	Type
158	1686.7879	1	-8.16	-0.0138	38.17	92.92	1	0.0	0	K.SGEGSQHPFGAMNIVR.T		89-104	CID
112	1042.4743	1	0.19	0.0002	34.67	51.29	1	0.0	0	K.WYENFQR.R		180-186	CID
218	1116.6077	1	-11.05	-0.0123	43.17	63.21	1	0.0	0	R.LAQNPLFWK.T		188-196	CID

Protein 2: Fermitin family homolog 1 OS=Homo sapiens OX=9606 GN=FERMT1 PE=1 SV=1

**Accession:**	FERM1_HUMAN	**Score:**	31.5
**Database:**	SwissProt	**Seq. Coverage [%]:**	1.9
**MW [kDa] / pI:**	77.4 / 5.9	**No. of Peptides:**	1

10	20	30	40	50	60	70	80	90	100
MLSSTDFTFA	SWELVVRVDH	PNEEQQKDVT	LRVSGDLHVG	GVMLKLVEQI	NISQDWSDFA	LWWEQKHCWL	LKTHWTLDKY	GVQADAKLLF	TPQHKMLRLR
110	120	130	140	150	160	170	180	190	200
LPNLKMVRLR	VSFSAVVFKA	VSDICKILNI	RRSEELSLLK	PSGDYFKKKK	KKDKNNKEPI	IEDILNLESS	PTASGSSVSP	GLYSKTMTPI	YDPINGTPAS
210	220	230	240	250	260	270	280	290	300
STMTWFSDSP	LTEQNCSILA	FSQPPQSPEA	LADMYQPRSL	VDKAKLNAGW	LDSSRSLMEQ	GIQEDEQLLL	RFKYYSFFDL	NPKYDAVRIN	QLYEQARWAI
310	320	330	340	350	360	370	380	390	400
LLEEIDCTEE	EMLIFAALQY	HISKLSLSAE	TQDFAGESEV	DEIEAALSNL	EVTLEGGKAD	SLLEDITDIP	KLADNLKLFR	PKKLLPKAFK	QYWFIFKDTS
410	420	430	440	450	460	470	480	490	500
IAYFKNKELE	QGEPLEKLNL	RGCEVVPDVN	VAGRKFGIKL	LIPVADGMNE	MYLRCDHENQ	YAQWMAACML	ASKGKTMADS	SYQPEVLNIL	SFLRMKNRNS
510	520	530	540	550	560	570	580	590	600
ASQVASSLEN	MDMNPECFVS	PRCAKRHKSK	QLAARILEAH	QNVAQMPLVE	AKLRFIQAWQ	SLPEFGLTYY	LVRFKGSKKD	DILGVSYNRL	IKIDAATGIP
610	620	630	640	650	660	670	680		
VTTWRFTNIK	QWNVNWETRQ	VVIEFDQNVF	TAFTCLSADC	KIVHEYIGGY	IFLSTRSKDQ	NETLDEDLFH	KLTGGQD		

MS/MS Peptide Matches

Cmpd.	m/z meas.	z	Δ m/z [ppm]	Δ m/z [Da]	Rt [min]	Score	No. of Cmpds.	Site [%]	P	Sequence	Modification	Range	Type
50	1400.7292	1	-17.16	-0.0240	28.83	31.48	1	0.0	0	K.IDAATGIPVTTWR.F		593-605	CID

Protein 3: Ribosome-binding protein 1 OS=Homo sapiens OX=9606 GN=RRBP1 PE=1 SV=5

**Accession:**	RRBP1_HUMAN	**Score:**	27.3
**Database:**	SwissProt	**Seq. Coverage [%]:**	0.7
**MW [kDa] / pI:**	152.4 / 8.7	**No. of Peptides:**	1

10	20	30	40	50	60	70	80	90	100
MDIYDTQTLG	VVVFGGFMVV	SAIGIFLVST	FSMKETSYEE	ALANQRKEMA	KTHHQKVEKK	KKEKTVEKKG	KTKKKEEKPN	GKIPDHDPAP	NVTVLLREPV
110	120	130	140	150	160	170	180	190	200
RAPAVAVAPT	PVQPPIIVAP	VATVPAMPQE	KLASSPKDKK	KKEKKVAKVE	PAVSSVVNSI	QVLTSKAAIL	ETAPKEVPMV	VVPPVGAKGN	TPATGTTQGK
210	220	230	240	250	260	270	280	290	300
KAEGTQNQSK	KAEGAPNQGR	KAEGTPNQGK	KTEGTPNQGK	KAEGTPNQGK	KAEGTPNQGK	KAEGAQNQGK	KVDTTPNQGK	KVEGAPTQGR	KAEGAQNQAK
310	320	330	340	350	360	370	380	390	400
KVEGAQNQGK	KAEGAQNQGK	KGEGAQNQGK	KAEGAQNQGK	KAEGAQNQGK	KAEGAQNQGK	KAEGAQNQGK	KAEGAQNQGK	KAEGAQNQGK	KVEGAQNQGK
410	420	430	440	450	460	470	480	490	500
KAEGAQNQGK	KAEGAQNQGK	KAEGAQNQGK	KAEGAQNQGK	KAEGAQNQGK	KAEGAQNQGK	KAEGAQNQGK	KVEGAQNQGK	KAEGAQNQGK	KAEGAQNQGK
510	520	530	540	550	560	570	580	590	600
KAEGAQNQGQ	KGEGAQNQGK	KTEGAQGKKA	ERSPNQGKKG	EGAPIQGKKA	DSVANQGTKV	EGITNQGKKA	EGSPSEGKKA	EGSPNQGKKA	DAAANQGKKT
610	620	630	640	650	660	670	680	690	700
ESASVQGRNT	DVAQSPEAPK	QEAPAKKKSG	SKKKGEPGPP	DADGPLYLPY	KTLVSTVGSM	VFNEGEAQRL	IEILSEKAGI	IQDTWHKATQ	KGDPVAILKR
710	720	730	740	750	760	770	780	790	800
QLEEKEKLLA	TEQEDAAVAK	SKLRELNKEM	AAEKAKAAAG	EAKVKKQLVA	REQEITAVQA	RMQASYREHV	KEVQQLQGKI	RTLQEQLENG	PNTQLARLQQ
810	820	830	840	850	860	870	880	890	900
ENSILRDALN	QATSQVESKQ	NAELAKLRQE	LSKVSKELVE	KSEAVRQDEQ	QRKALEAKAA	AFEKQVLQLQ	ASHRESEEAL	QKRLDEVSRE	LCHTQSSHAS
910	920	930	940	950	960	970	980	990	1000
LRADAEKAQE	QQQQMAELHS	KLQSSEAEVR	SKCEELSGLH	GQLQEARAEN	SQLTERIRSI	EALLEAGQAR	DAQDVQASQA	EADQQQTRLK	ELESQVSGLE
1010	1020	1030	1040	1050	1060	1070	1080	1090	1100
KEAIELREAV	EQQKVKNNDL	REKNWKAMEA	LATAEQACKE	KLLSLTQAKE	ESEKQLCLIE	AQTMEALLAL	LPELSVLAQQ	NYTEWLQDLK	EKGPTLLKHP
1110	1120	1130	1140	1150	1160	1170	1180	1190	1200
PAPAEPSSDL	ASKLREAEET	QSTLQAECDQ	YRSILAETEG	MLRDLQKSVE	EEEQVWRAKV	GAAEEELQKS	RVTVKHLEEI	VEKLKGELES	SDQVREHTSH
1210	1220	1230	1240	1250	1260	1270	1280	1290	1300
LEAELEKHMA	AASAECQNYA	KEVAGLRQLL	LESQSQLDAA	KSEAQKQSDE	LALVRQQLSE	MKSHVEDGDI	AGAPASSPEA	PPAEQDPVQL	KTQLEWTEAI
[illegible]	[illegible]	[illegible]	[illegible]	[illegible]	[illegible]	[illegible]	[illegible]	[illegible]	[illegible]

MS/MS Peptide Matches

Cmpd.	m/z meas.	z	Δ m/z [ppm]	Δ m/z [Da]	Rt [min]	Score	No. of Cmpds.	Site [%]	P	Sequence	Modification	Range	Type
180	1142.7105	1	18.65	0.0213	41.00	27.28	1	0.0	1	R.AATRLQELLK.T		1366-1375	CID
