# Supplementary material for: The Infantile Leukoencephalopathy-Associated Mutation of C11ORF73/HIKESHI Proteins Generates De Novo Interactive Activity with Filamin A, Inhibiting Oligodendroglial Cell Morphological Differentiation
Source: Medicines (Basel). 2021 Feb 1;8(2):9. doi: 10.3390/medicines8020009 (PMC7912763; doi:10.3390/medicines8020009)
Supplement: Supplementary file 1 [file medicines-08-00009-s001.zip › Figure S12.pdf]

Protein Report
LC-MALDI_OtherMammals_Mascot_2020-01-17 11:09:35

Project Info

Name: 200115-hikeshi-wt **Date:** Jan 15, 2020

Sample Info & Protocols

Name: 200116-Hikeshi-C4S
Date: Jan 16, 2020

Search Result Info

Search Result: LC-MALDI_OtherMammals_Mascot_2020-01-17 11:09:35
Location: /200115-hikeshi-wt/200116-Hikeshi-C4S/200116-Hikeshi-C4S
Search Method: LC-MALDI_OtherMammals **Version:**
Search Engine(s): Mascot, 2.6.2 **Ident. Compound(s):** 5/1564
Database(s): SwissProt, SwissProt_2019_07.fasta
Note:

Results

Protein 1: Filamin-A OS=Homo sapiens OX=9606 GN=FLNA PE=1 SV=4

**Accession:**	FLNA_HUMAN	**Score:**	76.2
**Database:**	SwissProt	**Seq. Coverage [%]:**	0.9
**MW [kDa] / pI:**	280.6 / 5.7	**No. of Peptides:**	2

10	20	30	40	50	60	70	80	90	100
MSSSHSRAGQ	SAAGAAPGGG	VDTRDAEMPA	TEKDLAEDAP	WKKIQQNTFT	RWCNEHLKCV	SKRIANLQTD	LSDGLRLIAL	LEVLSQKKMH	RKHNQRPTFR
110	120	130	140	150	160	170	180	190	200
QMQLENVSVA	LEFLDRESIK	LVSIDSKAIV	DGNLKLILGL	IWTLILHYSI	SMPMWDEEED	EEAKKQTPKQ	RLLGWIQNKL	PQLPITNFSR	DWQSGRALGA
210	220	230	240	250	260	270	280	290	300
LVDSCAPGLC	PDWDSWDASK	PVTNAREAMQ	QADDWLGIPQ	VITPEEIVDP	NVDEHSVMTY	LSQFPKAKLK	PGAPLRPKLN	PKKARAYGPG	IEPTGNMVKK
310	320	330	340	350	360	370	380	390	400
RAEFTVETRS	AGQGEVLVYV	EDPAGHQEEA	KVTANNDKNR	TFSVWYVPEV	TGTHKVTVLF	AGQHIAKSPF	EVYVDKSQGD	ASKVTAQGPG	LEPSGNIANK
410	420	430	440	450	460	470	480	490	500
TTYFEIFTAG	AGTGEVEVVI	QDPMGQKGTV	EPQLEARGDS	TYRCSYQPTM	EGVHTVHVTF	AGVPIPRSPY	TVTVGQACNP	SACRAVGRGL	QPKGVRVKET
510	520	530	540	550	560	570	580	590	600
ADFKVYTKGA	GSGELKVTVK	GPKGEERVKQ	KDLGDGVYGF	EYYPMVPGTY	IVTITWGGQN	IGRSPFEVKV	GTECGNQKVR	AWGPGLEGGV	VGKSADFVVE
610	620	630	640	650	660	670	680	690	700
AIGDDVGTLG	FSVEGPSQAK	IECDDKGDGS	CDVRYWPQEA	GEYAVHVLCN	SEDIRLSPFM	ADIRDAPQDF	HPDRVKARGP	GLEKTGVAVN	KPAEFTVDAK
710	720	730	740	750	760	770	780	790	800
HGGKAPLRVQ	VQDNEGCPVE	ALVKDNGNGT	YSCSYVPRKP	VKHTAMVSWG	GVSIPNSPFR	VNVGAGSHPN	KVKVYGPGVA	KTGLKAHEPT	YFTVDCAEAG
810	820	830	840	850	860	870	880	890	900
QGDVSIGIKC	APGVVGPAEA	DIDFDIIRND	NDTFTVKYTP	RGAGSYTIMV	LFADQATPTS	PIRVKVEPSH	DASKVKAEGP	GLSRTGVELG	KPTHFTVNAK
910	920	930	940	950	960	970	980	990	1000
AAGKGKLDVQ	FSGLTKGDAV	RDVDIIDHHD	NTYTVKYTPV	QQGPVGVNVT	YGGDPIPKSP	FSVAVSPSLD	LSKIKVSGLG	EKVDVGKDQE	FTVKSKGAGG
1010	1020	1030	1040	1050	1060	1070	1080	1090	1100
QGKVASKIVG	PSGAAVPCKV	EPGLGADNSV	VRFLPREEGP	YEVEVTYDGV	PVPGSPFPLE	AVAPTKPSKV	KAFGPGLQGG	SAGSPARFTI	DTKGAGTGGL
1110	1120	1130	1140	1150	1160	1170	1180	1190	1200
GLTVEGPCEA	QLECLDNGDG	TCSVSYVPTE	PGDYNINILF	ADTHIPGSPF	KAHVVPCFDA	SKVKCSGPGL	ERATAGEVGQ	FQVDCSSAGS	AELTIEICSE
1210	1220	1230	1240	1250	1260	1270	1280	1290	1300
AGLPAEVYIQ	DHGDGTHTIT	YIPLCPGAYT	VTIKYGGQPV	PNFPSKLQVE	PAVDTSGVQC	YGPGIEGQGV	FREATTEFSV	DARALTQTGG	PHVKARVANP
[illegible]	[illegible]	[illegible]	[illegible]	[illegible]	[illegible]	[illegible]	[illegible]	[illegible]	[illegible]

MS/MS Peptide Matches

Cmpd.	m/z meas.	z	Δ m/z [ppm]	Δ m/z [Da]	Rt [min]	Score	No. of Cmpds.	Site [%]	P	Sequence	Modification	Range	Type
1090	1226.6485	1	-3.50	-0.0043	37.83	45.91	1	0.0	0	R.AWGPGLEGGVVGK.S		581-593	CID
267	1108.6142	1	2.95	0.0033	25.17	30.3	1	0.0	0	R.ALTQTGGPHVK.A		1284-1294	CID

Protein 2: RNA-binding protein 10 OS=Homo sapiens OX=9606 GN=RBM10 PE=1 SV=3

**Accession:**	RBM10_HUMAN	**Score:**	69.7
**Database:**	SwissProt	**Seq. Coverage [%]:**	1.6
**MW [kDa] / pI:**	103.5 / 5.7	**No. of Peptides:**	1

BRUKER

10	20	30	40	50	60	70	80	90	100
MEYERRGGRG	DRTGRYGATD	RSQDDGGENR	SRDHDYRDMD	YRSYPREYGS	QEGKHDYDDS	SEEQSAEDSY	EASPGSETQR	RRRRRHRHSP	TGPPGFPRDG
110	120	130	140	150	160	170	180	190	200
DYRDQDYRTE	QGEEEEEEED	EEEEEKASNI	VMLRMLPQAA	TEDDIRGQLQ	SHGVQAREVR	LMRNKSSGQS	RGFAFVEFSH	LQDATRWMEA	NQHSLNILGQ
210	220	230	240	250	260	270	280	290	300
KVSMHYSDPK	PKINEDWLCN	KCGVQNFKRR	EKCFKCGVPK	SEAEQKLPLG	TRLDQQTLPL	GGRELSQGLL	PLPQPYQAQG	VLASQALSQG	SEPSSENAND
310	320	330	340	350	360	370	380	390	400
TIILRNLNPH	STMDSILGAL	APYAVLSSSN	VRVIKDKQTQ	LNRGFAFIQL	STIVEAAQLL	QILQALHPPL	TIDGKTINVE	FAKGSKRDMA	SNEGSRISAA
410	420	430	440	450	460	470	480	490	500
SVASTAIAAA	QWAISQASQG	GEGTWATSEE	PPVDYSYYQQ	DEGYGNSQGT	ESSLYAHGYL	KGTKGPGITG	TKGDPTGAGP	EASLEPGADS	VSMQAFSRAQ
510	520	530	540	550	560	570	580	590	600
PGAAPGIYQQ	SAEASSSQGT	AANSQSYTIM	SPAVLKSELQ	SPTHPSSALP	PATSPTAQES	YSQYPVPDVS	TYQYDETSGY	YYDPQTGLYY	DPNSQYYYNA
610	620	630	640	650	660	670	680	690	700
QSQQYLYWDG	ERRTYVPALE	QSADGHKETG	APSKEGKEKK	EKHKTKTAQQ	IAKDMERWAR	SLNKQKENFK	NSFQPISSLR	DDERRESATA	DAGYAILEKK
710	720	730	740	750	760	770	780	790	800
GALAERQHTS	MDLPKLASDD	RPSPPRGLVA	AYSGESDSEE	EQERGGPERE	EKLTDWQKLA	CLLCRRQFPS	KEALIRHQQL	SGLHKQNLEI	HRRAHLSENE
810	820	830	840	850	860	870	880	890	900
LEALEKNDME	QMKYRDRAAE	RREKYGIPEP	PEPKRRKYGG	ISTASVDFEQ	PTRDGLGSDN	IGSRMLQAMG	WKEGSGLGRK	KQGIVTPIEA	QTRVRGSGLG
910	920	930	940						
ARGSSYGVTS	TESYKETLHK	TMVTRFNEAQ							

MS/MS Peptide Matches

Cmpd.	m/z meas.	z	Δ m/z [ppm]	Δ m/z [Da]	Rt [min]	Score	No. of Cmpds.	Site [%]	P	Sequence	Modification	Range	Type
1299	1620.7583	1	-24.80	-0.0402	39.83	69.72	1	0.0	1	R.MLQAMGWKEGSGLGR.K		865-879	CID

Protein 3: Protein phosphatase 1B OS=Homo sapiens OX=9606 GN=PPM1B PE=1 SV=1

**Accession:**	PPM1B_HUMAN	**Score:**	37.9
**Database:**	SwissProt	**Seq. Coverage [%]:**	1.9
**MW [kDa] / pI:**	52.6 / 5.0	**No. of Peptides:**	1

10	20	30	40	50	60	70	80	90	100
MGAFLDKPKT	EKHNAHGAGN	GLRYGLSSMQ	GWRVEMEDAH	TAVVGIPHGL	EDWSFFAVYD	GHAGSRVANY	CSTHLLEHIT	TNEDFRAAGK	SGSALELSVE
**110**	**120**	**130**	**140**	**150**	**160**	**170**	**180**	**190**	**200**
NVKNGIRTGF	LKIDEYMRNF	SDLRNGMDRS	GSTAVGVMIS	PKHIYFINCG	DSRAVLYRNG	QVCFSTQDHK	PCNPREKERI	QNAGGSVMIQ	RVNGSLAVSR
**210**	**220**	**230**	**240**	**250**	**260**	**270**	**280**	**290**	**300**
ALGDYDYKCV	DGKGPTEQLV	SPEPEVYEIL	RAEEDEFIIL	ACDGIWDVMS	NEELCEYVKS	RLEVSDDLEN	VCNWVVDTCL	HKGSRDNMSI	VLVCFSNAPK
**310**	**320**	**330**	**340**	**350**	**360**	**370**	**380**	**390**	**400**
VSDEAVKKDS	ELDKHLESRV	EEIMEKSGEE	GMPDLAHVMR	ILSAENIPNL	PPGGGLAGKR	NVIEAVYSRL	NPHRESDGAS	DEAEESGSQG	KLVEALRQMR
**410**	**420**	**430**	**440**	**450**	**460**	**470**	**480**		
INHRGNYRQL	LEEMLTSYRL	AKVEGEESPA	EPAATATSSN	SDAGNPVTMQ	ESHTESESGL	AELDSSNEDA	GTKMSGEKI		

MS/MS Peptide Matches

Cmpd.	m/z meas.	z	Δ m/z [ppm]	Δ m/z [Da]	Rt [min]	Score	No. of Cmpds.	Site [%]	P	Sequence	Modification	Range	Type
1012	1050.5371	1	-19.74	-0.0207	35.83	37.87	1	0.0	0	R.NVIEAVYSR.L		361-369	CID

Protein 4: Probable tRNA (uracil-O(2)-)-methyltransferase OS=Homo sapiens OX=9606 GN=TRMT44 PE=1 SV=2

**Accession:**	TRM44_HUMAN	**Score:**	32.9
**Database:**	SwissProt	**Seq. Coverage [%]:**	1.1
**MW [kDa] / pI:**	84.6 / 7.0	**No. of Peptides:**	1

10	20	30	40	50	60	70	80	90	100
MAEVGRTGIS	YPGALLPQGF	WAAVEVWLER	PQVANKRLCG	ARLEARWSAA	LPCAEARGPG	TSAGSEQKER	GPGPGQGSPG	GGPGPRSLSG	PEQGTACCEL
110	120	130	140	150	160	170	180	190	200
EEAQGQCQQE	EAQREAASVP	LRDSGHPGHA	EGREGDFPAA	DLDSLWEDFS	QSLARGNSEL	LAFLTSSGAG	SQPEAQRELD	VVLRTVIPKT	SPHCPLTTPR
210	220	230	240	250	260	270	280	290	300
REIVVQDVLN	GTITFLPLEE	DDEGNLKVKM	SNVYQIQLSH	SKEEWFISVL	IFCPERWHSD	GIVYPKPTWL	GEELLAKLAK	WSVENKKSDF	KSTLSLISIM
310	320	330	340	350	360	370	380	390	400
KYSKAYQELK	EKYKEMVKVW	PEVTDPEKFV	YEDVAIAAYL	LILWEEERAE	RRLTARQSFV	DLGCGNGLLV	HILSSEGHPG	RGIDVRRRKI	WDMYGPQTQL
410	420	430	440	450	460	470	480	490	500
EEDAITPNDK	TLFPDVDWLI	GNHSDELTPW	IPVIAARSSY	NCRFFVLPCC	FFDFIGRYSR	RQSKKTQYRE	YLDFIKEVGF	TCGFHVDEDC	LRIPSTKRVC
510	520	530	540	550	560	570	580	590	600
LVGKSRTYPS	SREASVDEKR	TQYIKSRRGC	PVSPPGWELS	PSPRWVAAGS	AGHCDGQQAL	DARVGCVTRA	WAAEHGAGPQ	AEGPWLPGFH	PREKAERVRN
610	620	630	640	650	660	670	680	690	700
CAALPRDFID	QVVLQVANLL	LGGKQLNTRS	SRNGSLKTWN	GGESLSLAEV	ANELDTETLR	RLKRECGGLQ	TLLRNSHQVF	QVVNGRVHIR	DWREETLWKT
710	720	730	740	750	760				
KQPEAKQRLL	SEACKTRLCW	FFMHHPDGCA	LSTDCCPFAH	GPAELRPPRT	TPRKKIS				

MS/MS Peptide Matches

Cmpd.	m/z meas.	z	Δ m/z [ppm]	Δ m/z [Da]	Rt [min]	Score	No. of Cmpds.	Site [%]	P	Sequence	Modification	Range	Type
673	842.4784	1	6.36	0.0054	31.67	32.87	1	0.0	0	R.EAASVPLR.D		115-122	CID

Protein 5: Centrosome-associated protein 350 OS=Homo sapiens OX=9606 GN=CEP350 PE=1 SV=1

Accession: CE350_HUMAN
Database: SwissProt
MW [kDa] / pI: 350.7 / 6.0
Modification(s): Phospho

Score: 32.2
Seq. Coverage [%]: 0.0
No. of Peptides: 0

10	20	30	40	50	60	70	80	90	100
MRSSKSKEVP	LPNPRNSQSK	DTVQADITTS	WDALSQTKAA	LRHIENKLEV	APTSTAVCDS	VMDTKKSSTS	ATRKISRKDG	RYLDDSWVNA	PISKSTKSRK
110	120	130	140	150	160	170	180	190	200
EKSRSPLRAT	TLESNVKKNN	RVEFREPLVS	YREIHGAPSN	FSSSHLESKH	VYCVDVNEEK	TESGNWMIGS	REERNIRSCD	FESSQSSVIN	DTVVRFLNDR
210	220	230	240	250	260	270	280	290	300
PAIDALQNSE	CLIRMGASMR	TEEEMPNRTK	GSENNLKLSV	NNMAHDTDPK	ALRLTDSSPS	STSTSNSQRL	DILKRRQHDV	KLEKLKERIR	KQWEHSEETN
310	320	330	340	350	360	370	380	390	400
GRGQKLGHID	HPVMVVNVDN	SVTAKVRKVA	TAPPAPAYKG	FNPSETKIRT	PDGKVWQEAE	FQNMSRELYR	DLALHFADDI	SIKEKPAEKS	KEKKVVKPVR
410	420	430	440	450	460	470	480	490	500
KVQKVAQLSS	TECRTGSSHL	ISTSSWRDGQ	KLVKKILGPA	PRMEPKEQRT	ASSDRGGRER	TAKSGGHIGR	AESDPRLDVL	HRHLQRNSER	SRSKSRSENN
510	520	530	540	550	560	570	580	590	600
IKKLASSLPD	NKQEENTALN	KDFLPIEIRG	ILDDLQLDST	AHTAKQDTVE	LQNQKSSAPV	HAPRSHSPVK	RKPDKITANE	DPPVISKRRH	YDTDEVRQYI
610	620	630	640	650	660	670	680	690	700
VRQQEERKRK	QNEEKKAQKE	ATEQKNKRLQ	ELYRKQKEAF	TKVKNVPPSE	PSATRRLQET	YSKLLLEKTL	LEEPSHQHVT	QETQAKPGYQ	PSGESDKENK
710	720	730	740	750	760	770	780	790	800
VQERPPSASS	SSDMSLSEPP	QPLARKDLME	STWMQPERLS	PQVHHSQPQP	FAGTAGSLLS	HLLSLEHVGI	LHKDFESILP	TRKNHNMASR	PLTFTPQPYV
810	820	830	840	850	860	870	880	890	900
TSPAAYTDAL	LKPSASQYKS	KLDRIEALKA	TAASLSSRIE	SEAKKLAGAS	INYGSAWNTE	YDVQQAPQED	GPWTKAVTPP	VKDDNEDVFS	ARIQKMLGSC
910	920	930	940	950	960	970	980	990	1000
VSHATFDDDL	PGVGNLSEFK	KLPEMIRPQS	AISSFRVRSP	GPKPEGLLAQ	LCKRQTDSSS	SDMQACSQDK	AKISLGSSID	SVSEGPLLSE	GSLSEEEGDQ
1010	1020	1030	1040	1050	1060	1070	1080	1090	1100
DGQPLLKVAE	ILKEKEFCPG	ERNSYEPIKE	FQKEAEKFLP	LFGHIGGTQS	KGPWEELAKG	SPHSVINIFT	KSYQLYGKGF	EDKLDRGTST	SRPLNATATP
1110	1120	1130	1140	1150	1160	1170	1180	1190	1200
LSGVSYEDDF	VSSPGTGTST	EKKSTLEPHS	TLSPQEDHSN	RKSAYDPSSV	DVTSQHSSGA	QSAASSRSST	SSKGKKGKKE	KTEWLDSFTG	NVQNSLLDEE
1210	1220	1230	1240	1250	1260	1270	1280	1290	1300
KAERGSHQGK	KSGTSSKLSV	KDFEQTLDTD	STLEDLSGHS	VSVSSDKGRS	QKTPTSPLSP	SSQKSLQFDV	AGTSSERSKS	SVMPPTITGF	KPNAPLTDLN
[illegible]	[illegible]	[illegible]	[illegible]	[illegible]	[illegible]	[illegible]	[illegible]	[illegible]	[illegible]
