# Supplementary material for: The Infantile Leukoencephalopathy-Associated Mutation of C11ORF73/HIKESHI Proteins Generates De Novo Interactive Activity with Filamin A, Inhibiting Oligodendroglial Cell Morphological Differentiation
Source: Medicines (Basel). 2021 Feb 1;8(2):9. doi: 10.3390/medicines8020009 (PMC7912763; doi:10.3390/medicines8020009)
Supplement: Supplementary file 1 [file medicines-08-00009-s001.zip › Figure S13.pdf]

Project Info

**Name:**	200115-hikeshi-wt	**Date:**	Jan 15, 2020

Sample Info & Protocols

**Name:**	200117-Hikeshi-V54L
**Date:**	Jan 17, 2020

Search Result Info

**Search Result:**	LC-MALDI_OtherMammals_Mascot_2020-01-17 15:16:27		
**Location:**	/200115-hikeshi-wt/200117-Hikeshi-V54L/200117-Hikeshi-V54L		
**Search Method:**	LC-MALDI_OtherMammals	**Version:**	
**Search Engine(s):**	Mascot, 2.6.2	**Ident. Compound(s):**	0/639
**Database(s):**	SwissProt, SwissProt_2019_07.fasta		
**Note:**			

Results
