# Supplementary material for: The Infantile Leukoencephalopathy-Associated Mutation of C11ORF73/HIKESHI Proteins Generates De Novo Interactive Activity with Filamin A, Inhibiting Oligodendroglial Cell Morphological Differentiation
Source: Medicines (Basel). 2021 Feb 1;8(2):9. doi: 10.3390/medicines8020009 (PMC7912763; doi:10.3390/medicines8020009)
Supplement: Supplementary file 1 [file medicines-08-00009-s001.zip › medicines-1035191-SUPP final-1-10.pdf]

Supplementary Materials: The Infantile Leukoencephalopathy-Associated Mutation of C11ORF73/HIKESHI Proteins Generates de novo Interactive Activity with Filamin A, Inhibiting Oligodendroglial Cell Morphological Differentiation

Kohei Hattori, Kenji Tago, Shiori Memezawa, Arisa Ochiai, Sui Sawaguchi, Yukino Kato, Takanari Sato, Kazuma Tomizuka, Hiroaki Ooizumi, Katsuya Ohbuchi, Kazushige Mizoguchi, Yuki Miyamoto and Junji Yamauchi

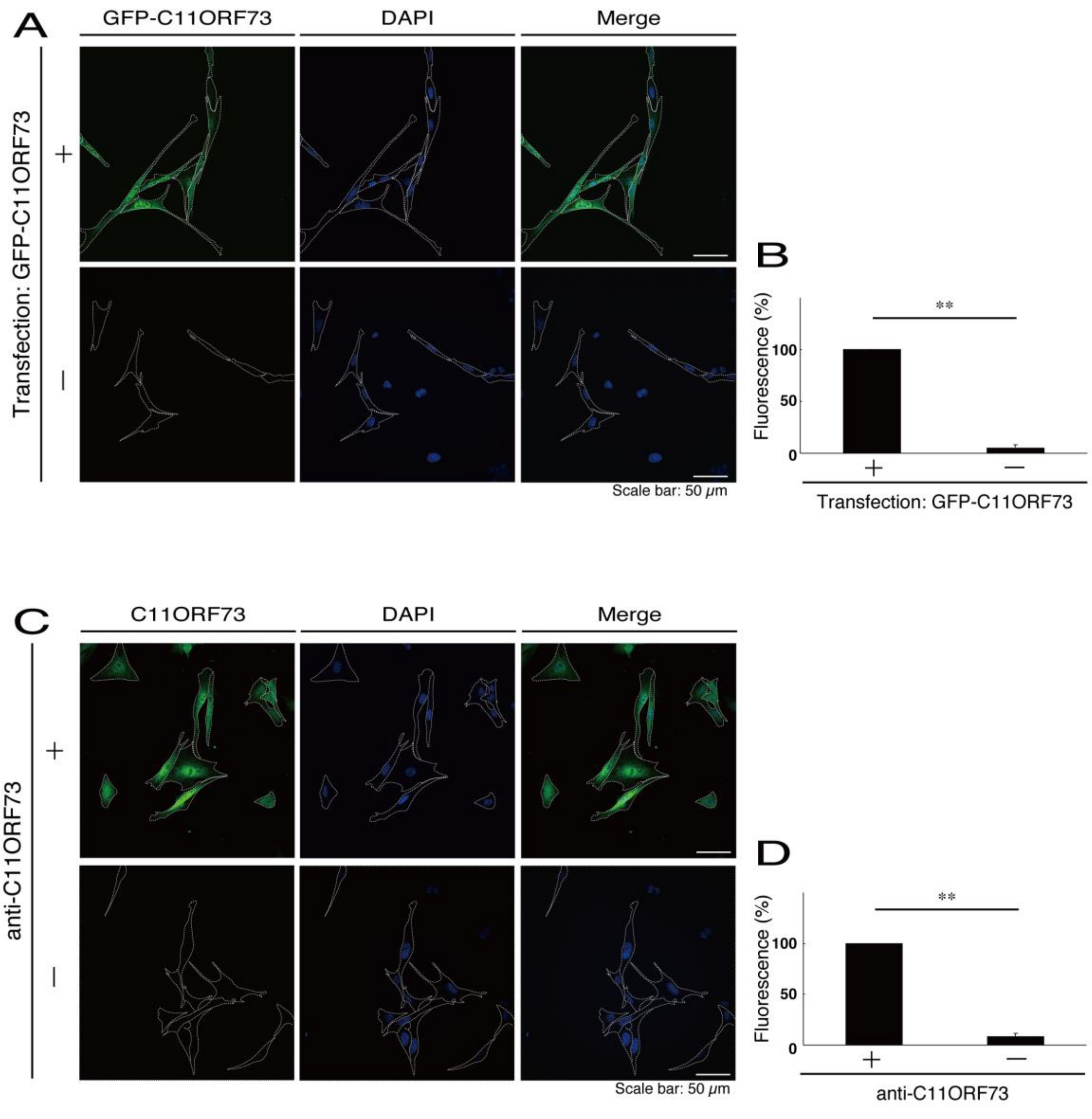

Figure S1. GFP-tagged C11ORF73 proteins exhibit similar localization to endogenous C11ORF73 ones. (**A**) FBD-102b cells, which were surrounded by dotted lines, were transfected with (+) or without (−, a pCMV5 vector) a plasmid encoding GFP-tagged C11ORF73 (green) and stained with DAPI (blue). (**B**) Graphs to compare green fluorescence intensities of cells transfected with or without a plasmid encoding GFP-tagged C11ORF73 are shown (**, $p < 0.01$ of Student's t-test; $n = 50$ cells in 3 fields). (**C**) FBD-102b cells were stained with (+) or without (−, a control rabbit antibody) an anti-C11ORF73 antibody in the presence of DAPI (blue). Secondary antibodies (green) were normally treated for both conditions. (**D**) Graphs to compare green fluorescence intensities with or without an anti-C11ORF73 antibody are shown (**, $p < 0.01$ of Student's t-test; $n = 25$ cells in total).

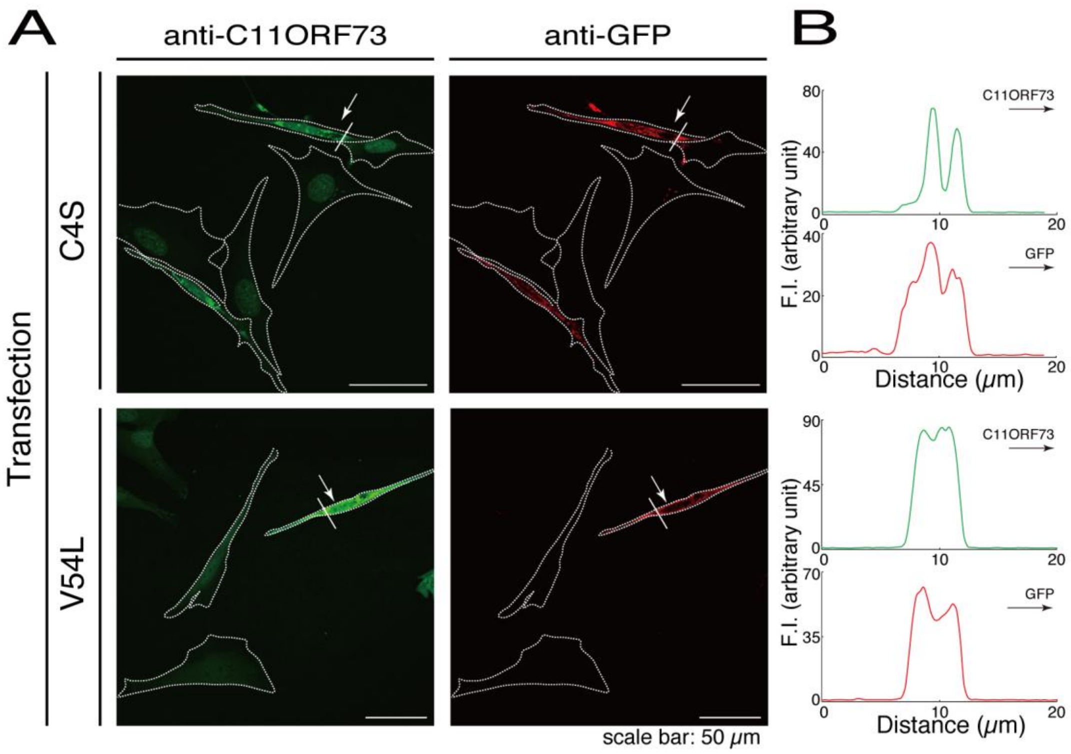

Figure S2. An anti-C11ORF73 antibody-stained C11ORF73 mutant proteins exhibit similar localization to an anti-GFP antibody-stained ones. (**A, B**) FBD-102b cells, which were transfected with a plasmid encoding GFP-tagged C11ORF73 (C4S or V54L), were stained with an antibody against C11ORF73 (green) or GFP (red). Graphs showing the fluorescence intensities (F.I., in an arbitrary unit) along the white lines can be seen in the panels.

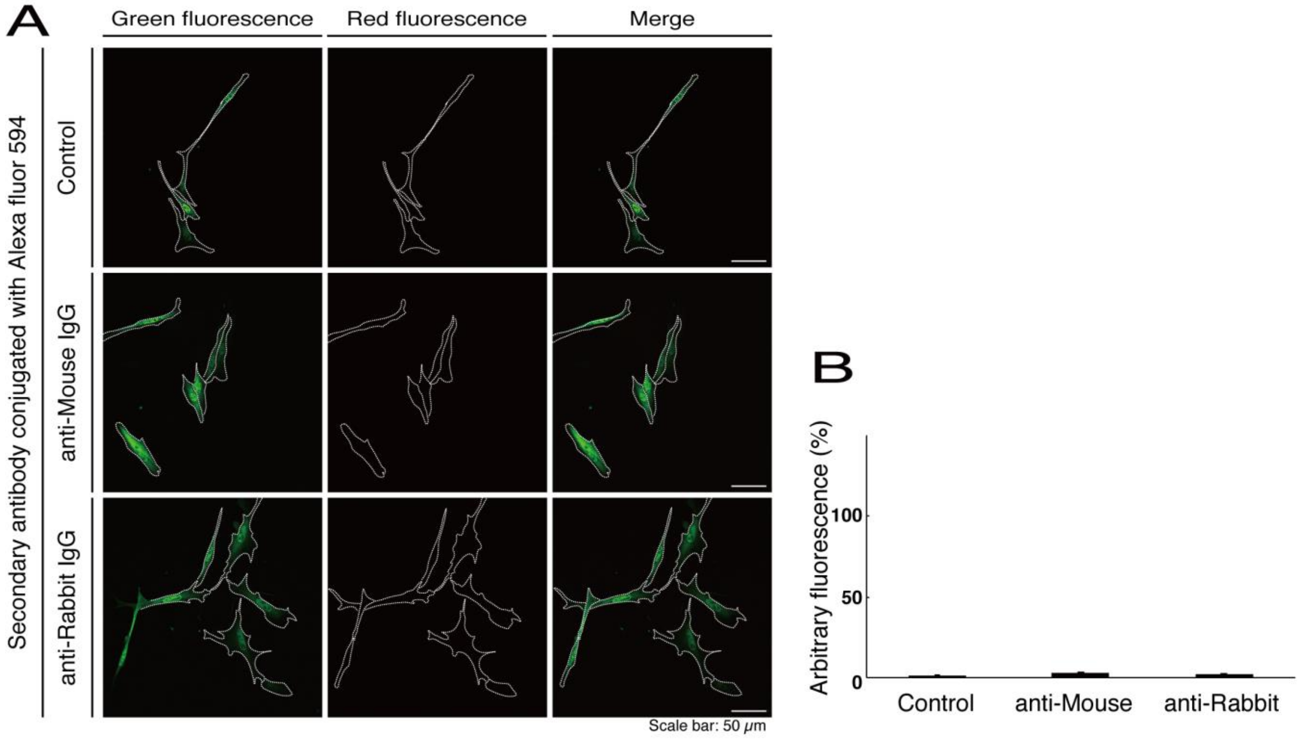

Figure S3. Secondary antibodies have no abilities to stain cells. (**A**) FBD-102b cells, which were surrounded by dotted lines, were transfected with a plasmid encoding GFP-tagged C11ORF73 (green) and stained with an anti-mouse or anti-rabbit secondary antibody or a control antibody (red) in the absence of the primary antibody. (**B**) Graphs to compare red fluorescence intensities are shown (n = 25 cells in total).

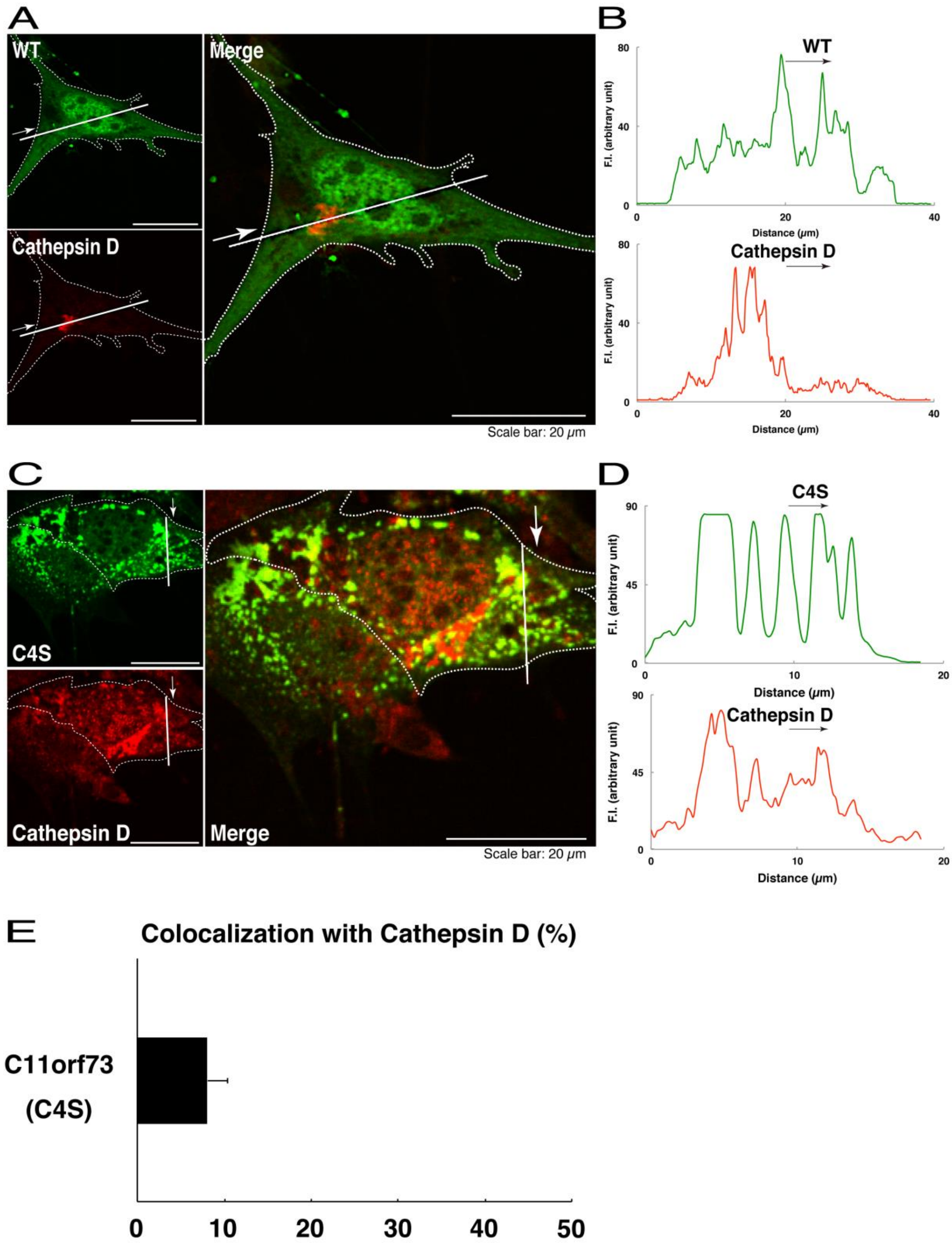

Figure S4. The C4S mutant proteins of C11ORF73 are colocalized with the Cathepsin D marker. (**A**) FBD-102b cells, which were surrounded by dotted lines, were transfected with a plasmid encoding wild type (WT) GFP-tagged C11ORF73 (green) and stained with the lysosome marker Cathepsin D (red). A scan plot was performed along the white line in the direction of the arrow in the image. (**B**) Graphs showing the fluorescence intensities (F.I., in an arbitrary unit) along the white lines can be seen in the panels. (**C**) Cells were transfected with a plasmid encoding the C4S mutant construct (green) and stained with the lysosome marker (red). A scan plot was performed along the white line in the direction of the arrow in the image. (**D**) Graphs showing the fluorescence intensities (F.I., in an arbitrary unit) along the white lines can be seen in the panels. (**E**) Merged percentages of mutant proteins with organelles (percentages of yellow-colored pixels/green-colored pixels) are shown in the graph (n = 3 independent experiments).

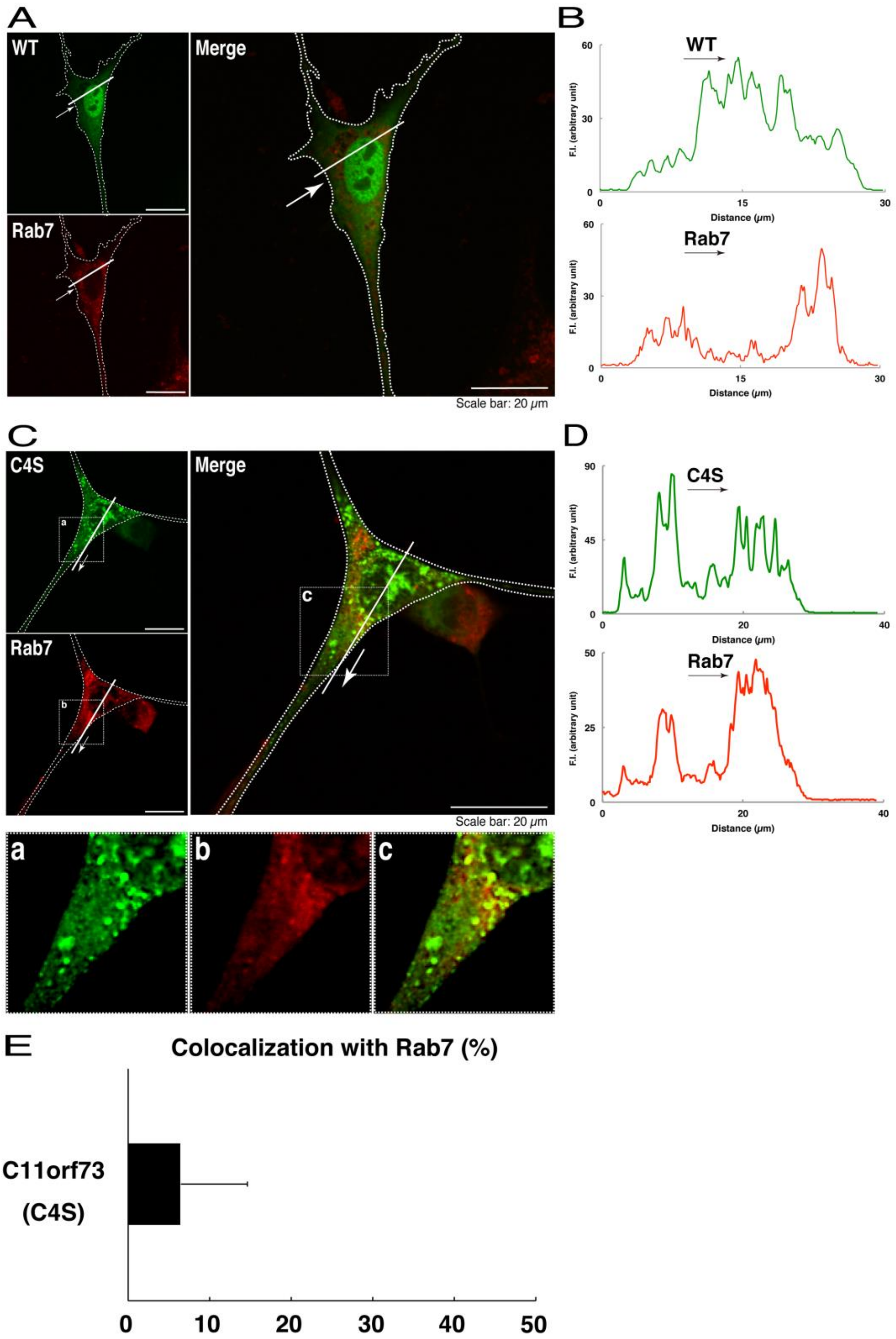

Figure S5. The C4S mutant proteins of C11ORF73 are not colocalized with Rab7. (**A**) FBD-102b cells, which were surrounded by dotted lines, were transfected with a plasmid encoding wild type (WT) GFP-tagged C11ORF73 (green) and stained with an anti-Rab7 antibody (red). A scan plot was performed along the white line in the direction of the arrow in the image. (**B**) Graphs showing the fluorescence intensities (F.I., in an arbitrary unit) along the white lines can be seen in the panels. (**C**) Cells were transfected with a plasmid encoding the C4S mutant construct (green) and stained with an anti-Rab7 antibody (red). Regions (a to c) within white squares are magnified in the below panels. A scan plot was performed along the white line in the direction of the arrow in the image. (**D**) Graphs showing the fluorescence intensities (F.I., in an arbitrary unit) along the white lines can be seen in the panels. (**E**) Merged percentages of mutant proteins with organelles (percentages of yellow-colored pixels/green-colored pixels) are shown in the graph (n = 3 independent experiments).

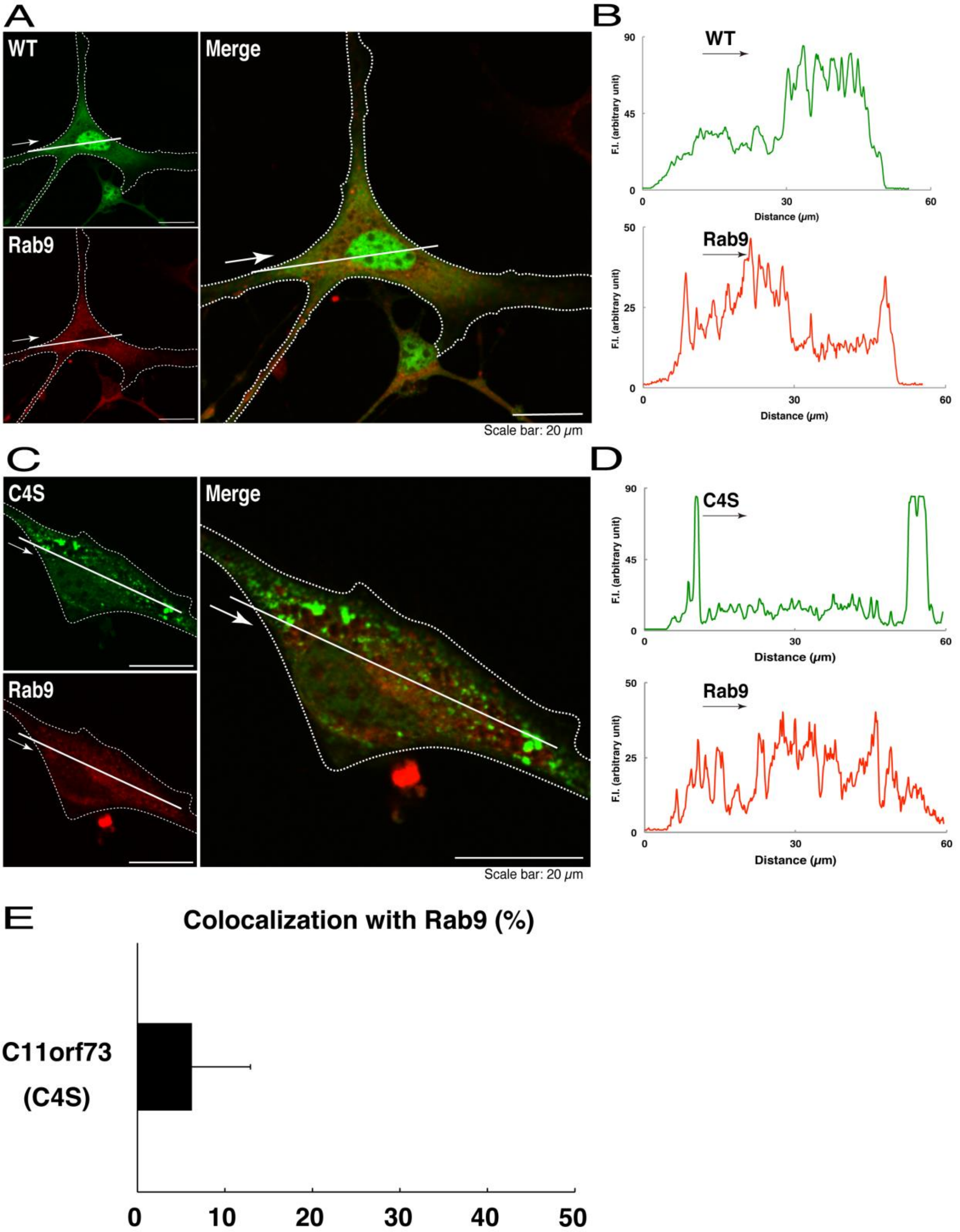

Figure S6. The C4S mutant proteins of C11ORF73 are not colocalized with Rab9. (**A**) FBD-102b cells, which were surrounded by dotted lines, were transfected with a plasmid encoding wild type (WT) GFP-tagged C11ORF73 (green) and stained with an anti-Rab9 antibody (red). A scan plot was performed along the white line in the direction of the arrow in the image. (**B**) Graphs showing the fluorescence intensities (F.I., in an arbitrary unit) along the white lines can be seen in the panels. (**C**) Cells were transfected with a plasmid encoding the C4S mutant construct (green) and stained with an anti-Rab9 antibody (red). A scan plot was performed along the white line in the direction of the arrow in the image. (**D**) Graphs showing the fluorescence intensities (F.I., in an arbitrary unit) along the white lines can be seen in the panels. (**E**) Merged percentages of mutant proteins with organelles (percentages of yellow-colored pixels/green-colored pixels) are shown in the graph (*n* = 3 independent experiments).

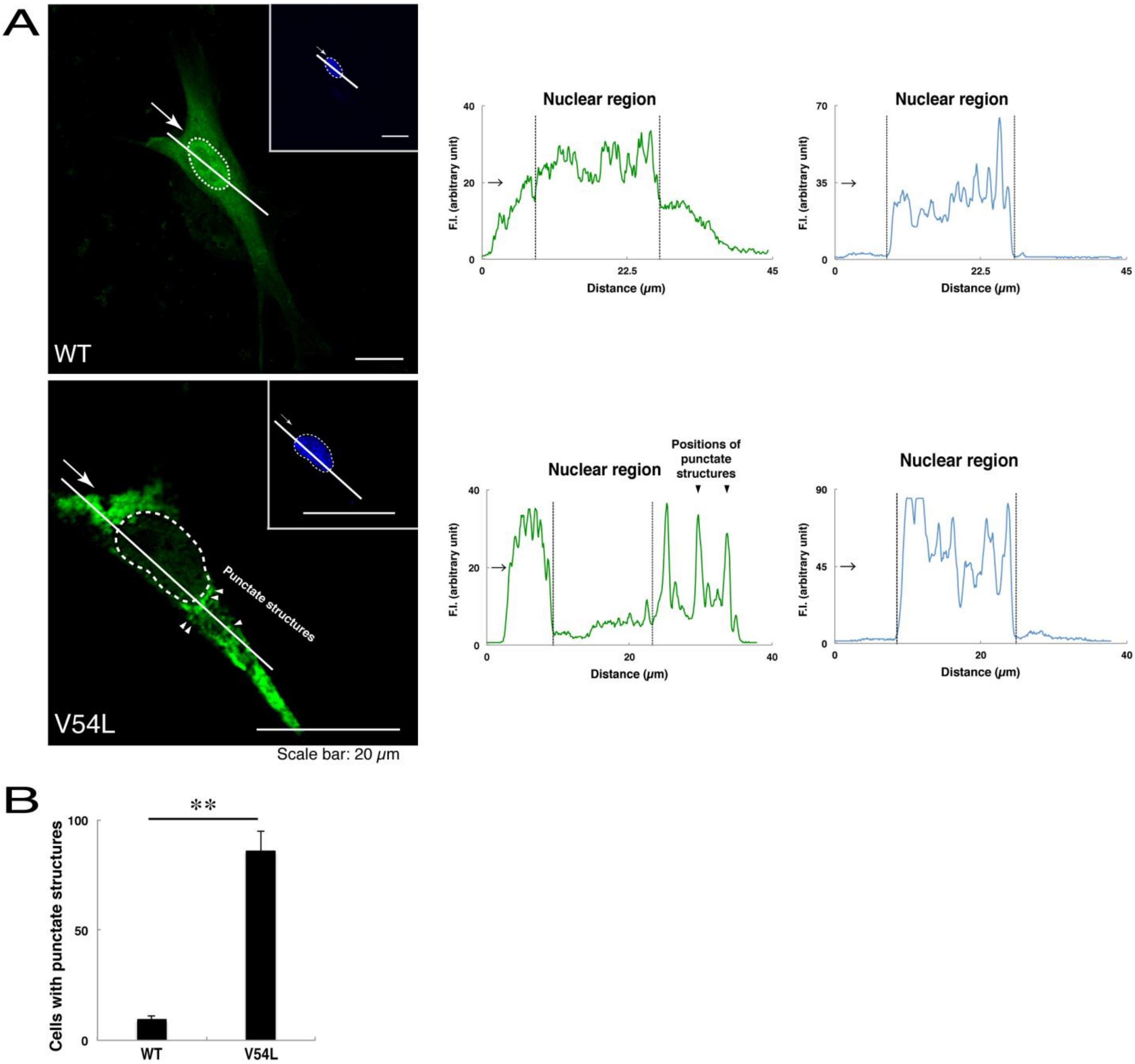

Figure S7. The V54L mutant proteins of C11ORF73 accumulate in punctate structures in cells. (**A**) FBD-102b cells were transfected with a plasmid encoding wild type (WT) or the V54L mutant construct of GFP-tagged C11ORF73 (green) and stained with DAPI (blue). A scan plot was performed along the white line in the direction of the arrow in the image. Graphs showing the fluorescence intensities (F.I., in an arbitrary unit) along the white lines can be seen in the right panels. Some punctate structures (indicated by arrowheads) and nuclear regions (surrounded by dotted lines) are also shown in the images and graphs. (**B**) Percentages of cells with punctate structures are statistically shown (**, $p < 0.01$ of Student's t-test; $n = 3$ fields).

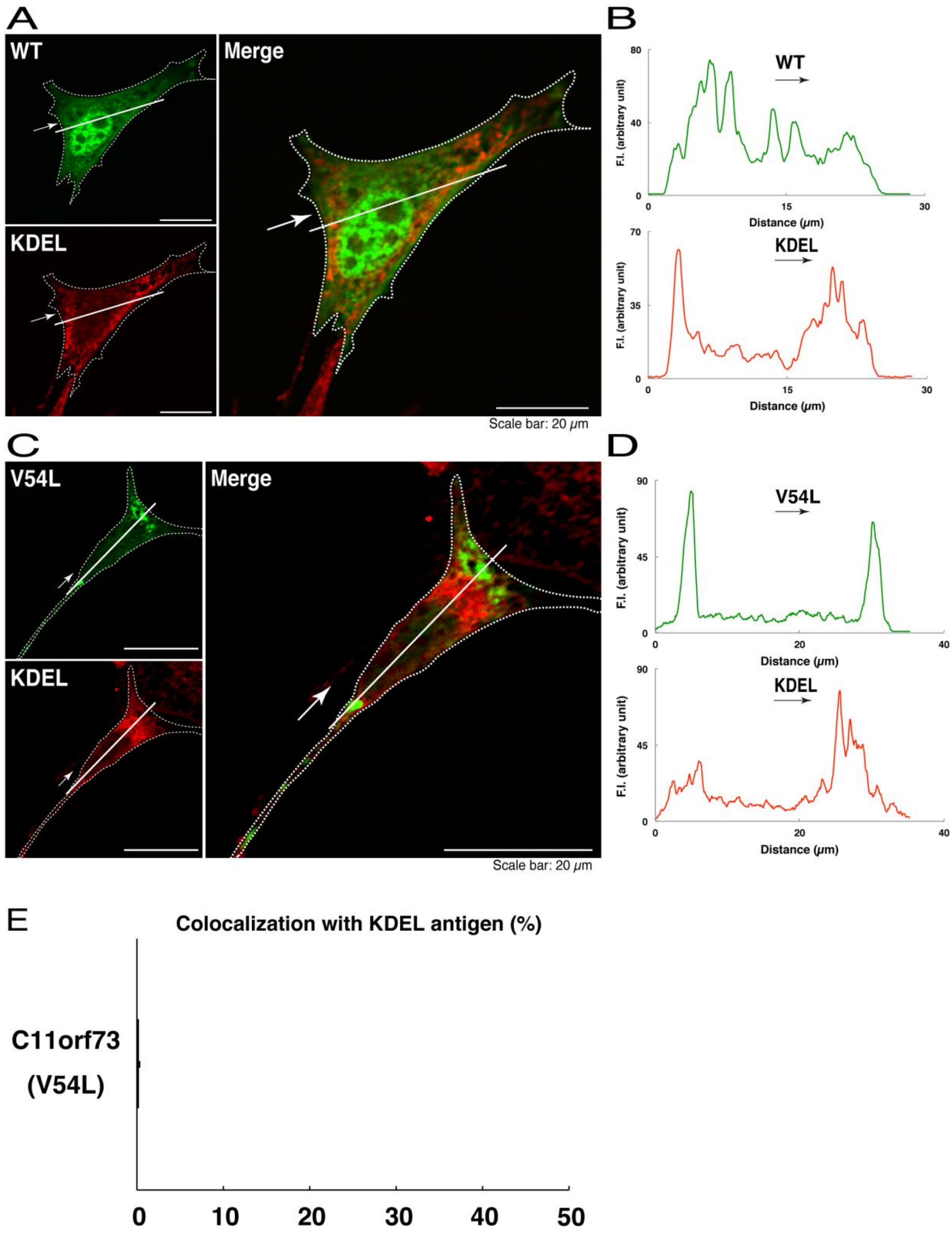

Figure S8. The V54L mutant proteins of C11ORF73 are colocalized with the KDEL antigen. (**A**) FBD-102b cells, which were surrounded by dotted lines, were transfected with a plasmid encoding wild type (WT) GFP-tagged C11ORF73 (green) and stained with an anti-KDEL antigen antibody (red). A scan plot was performed along the white line in the direction of the arrow in the image. (**B**) Graphs showing the fluorescence intensities (F.I., in an arbitrary unit) along the white lines can be seen in the panels. (**C**) Cells were transfected with a plasmid encoding the V54L mutant construct (green) and stained with an anti-KDEL antigen antibody (red). A scan plot was performed along the white line in the direction of the arrow in the image. (**D**) Graphs showing the fluorescence intensities (F.I., in an arbitrary unit) along the white lines can be seen in the panels. (**E**) Merged percentages of mutant proteins with organelles (percentages of yellow-colored pixels/green-colored pixels) are shown in the graph (*n* = 3 independent experiments).

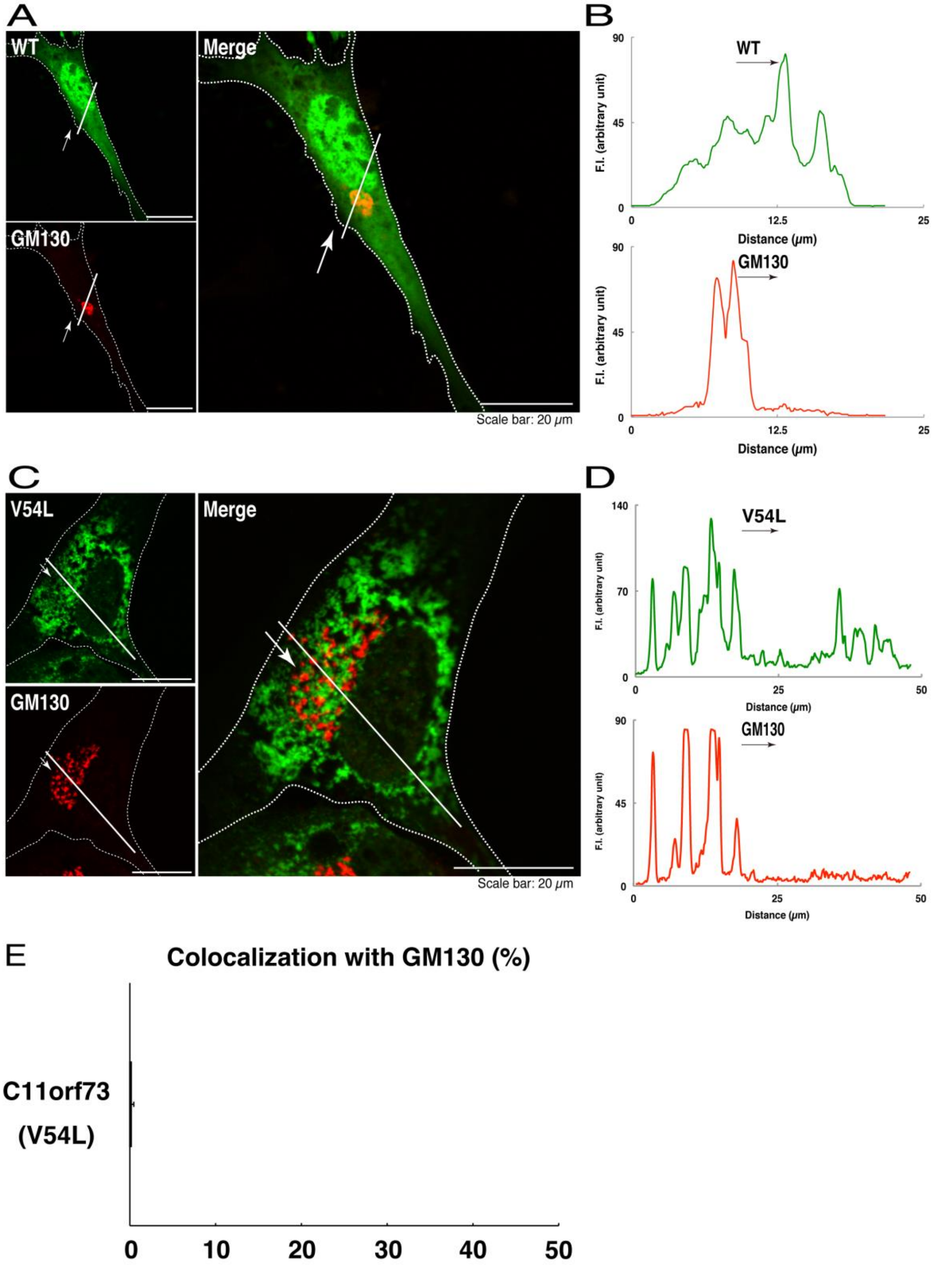

Figure S9. The V54L mutant proteins of C11ORF73 are not colocalized with GM130. (**A**) FBD-102b cells, which were surrounded by dotted lines, were transfected with a plasmid encoding wild type (WT) GFP-tagged C11ORF73 (green) and stained with an anti-GM130 antibody (red). A scan plot was performed along the white line in the direction of the arrow in the image. (**B**) Graphs showing the fluorescence intensities (F.I., in an arbitrary unit) along the white lines can be seen in the panels. (**C**) Cells were transfected with a plasmid encoding the V54L mutant construct (green) and stained with an anti-GM130 antibody (red). A scan plot was performed along the white line in the direction of the arrow in the image. (**D**) Graphs showing the fluorescence intensities (F.I., in an arbitrary unit) along the white lines can be seen in the panels. (**E**) Merged percentages of mutant proteins with organelles (percentages of yellow-colored pixels/green-colored pixels) are shown in the graph (n = 3 independent experiments).

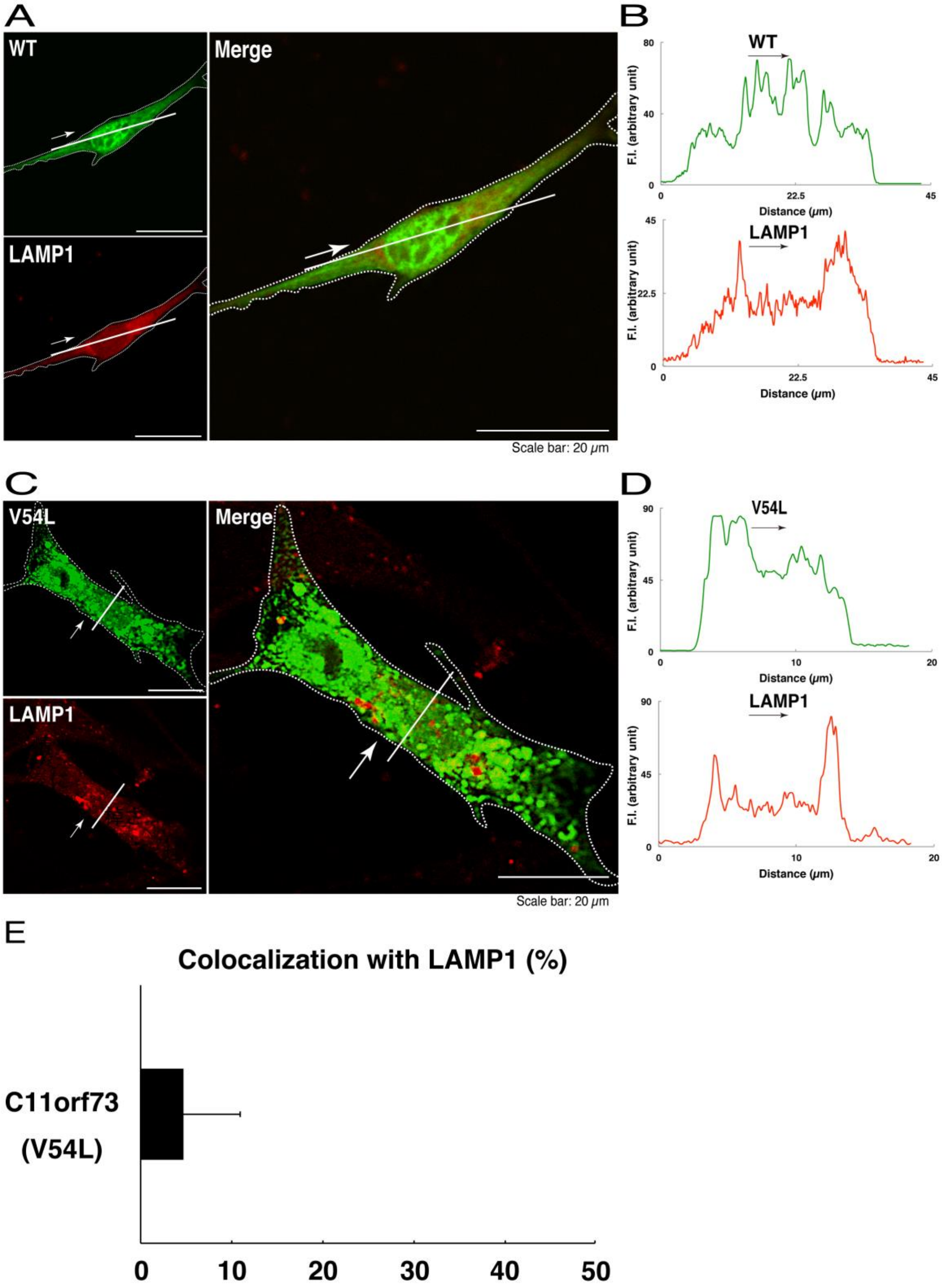

Figure S10. The V54L mutant proteins of C11ORF73 are not colocalized with LAMP1. (**A**) FBD-102b cells, which were surrounded by dotted lines, were transfected with a plasmid encoding wild type (WT) GFP-tagged C11ORF73 (green) and stained with an anti-LAMP1 antibody (red). A scan plot was performed along the white line in the direction of the arrow in the image. (**B**) Graphs showing the fluorescence intensities (F.I., in an arbitrary unit) along the white lines can be seen in the panels. (**C**) Cells were transfected with a plasmid encoding the V54L mutant construct (green) and stained with an anti-LAMP1 antibody (red). A scan plot was performed along the white line in the direction of the arrow in the image. (**D**) Graphs showing the fluorescence intensities (F.I., in an arbitrary unit) along the white lines can be seen in the panels. (**E**) Merged percentages of mutant proteins with organelles (percentages of yellow-colored pixels/green-colored pixels) are shown in the graph (*n* = 3 independent experiments).
